# A comprehensive review on the advancements and challenges in perovskite solar cell technology

**DOI:** 10.1039/d3ra07518d

**Published:** 2024-02-08

**Authors:** Muhammad Noman, Zeeshan Khan, Shayan Tariq Jan

**Affiliations:** a U.S. – Pakistan Center for Advanced Studies in Energy, University of Engineering & Technology Peshawar Pakistan muhammad.noman@uetpeshawar.edu.pk; b Department of Energy Engineering Technology, University of Technology Nowshera Pakistan

## Abstract

Perovskite solar cells (PSCs) have emerged as revolutionary technology in the field of photovoltaics, offering a promising avenue for efficient and cost-effective solar energy conversion. This review provides a comprehensive overview of the progress and developments in PSCs, beginning with an introduction to their fundamental properties and significance. Herein, we discuss the various types of PSCs, including lead-based, tin-based, mixed Sn–Pb, germanium-based, and polymer-based PSCs, highlighting their unique attributes and performance metrics. Special emphasis is given to halide double PSCs and their potential in enhancing the stability of PSCs. Charge transport layers and their significance in influencing the overall efficiency of solar cells are discussed in detail. The review also explores the role of tandem solar cells as a solution to overcome the limitations of single-junction solar cells, offering an integrated approach to harness a broader spectrum of sunlight. This review concludes with challenges associated with PSCs and perspective on the future potential of PSCs, emphasizing their role in shaping a sustainable energy landscape. Through this review readers will gain a comprehensive insight into the current state-of-the-art in PSC technology and the avenues for future research and development.

## Abstract 2

This review provides an overview of the progress & developments of PSCs, beginning with introduction to their fundamental properties & significance. It discusses the various types of PSCs, highlighting their unique attributes & performance metrics.

## Graphical abstract

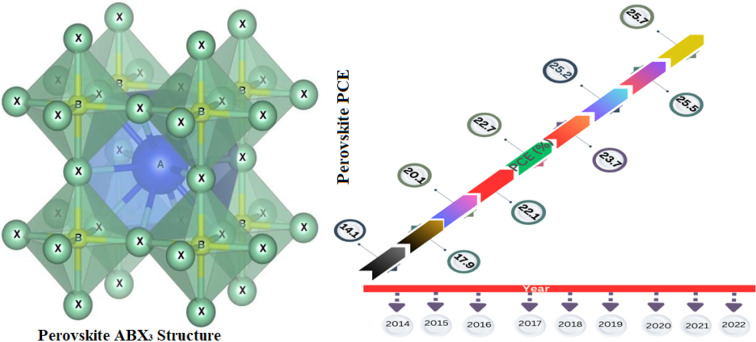

## Introduction

The non-renewable sources of fossil fuels is the main contributor for providing reliable energy throughout the world. The global energy demand rises steadily as the earth's population increases over time. The depletion of these non-renewable energy sources along with their environmental issues has caused major concerns. Owing to the discharge of carbon and other hazardous elements into the environment, the world is now dealing with a serious environmental problem. This has led to the utilization of alternative energy sources, especially renewable energy. In this context, solar energy is recognized as an essential renewable energy source due to its quantity, purity, and inexhaustibility. Furthermore, the use of solar energy does not harm the environment.^1^ Numerous methods have been developed in the last few years to capture and utilize solar energy, including solar heating, artificial photosynthesis, photovoltaics, photocatalytic water splitting, and solar architecture.^2,3^ Among the various solar technologies, photovoltaics (PV) has attracted major attention. This technology is based on the phenomenon known as photovoltaics, in which the photon energy of sunlight is directly converted into electrical energy through a device called a PV cell or solar cell. By switching to PV technology, the amount of pollutants in the physical environment and the release of poisonous gases can be reduced. According to reports, until 2050, there must be a decrease in carbon emissions of 25 000 GW for a sustainable environment.

Solar cell technology is often divided into three generations based on the materials used in the devices. Silicon wafer-based solar cells make up the first generation, whereas thin film-based solar cells make up the second generation. Similarly, the third-generation cells are comprised of organic, dye-sensitized, quantum dot, and perovskite materials. The PV market is dominated by the 1st and 2nd generation solar cells. However, these technologies are associated with the issues of high fabrication cost, complicated production procedures, and low efficiency. This has led researchers towards developing innovative, low-cost, and efficient materials for solar cells.

Numerous types of solar cells have been discovered thus far, including perovskite and organic cells and polycrystalline-silicon (mc-Si cells), single-crystalline silicon (c-Si cells), CIGS solar cells, CdTe-based solar cells, quantum dot-sensitized solar cells and polycrystalline-silicon (mc-Si cells).^4,5^ However, to realize the wide commercialization of solar cells, their low-cost manufacture and high conversion efficiency are important. To date, the first-generation silicon-based solar cells have been the most popular in the market because of their high-power conversion efficiency (PCE) of 25–26% and durability. However, they are hampered by their long fabrication time and high cost. Recently, perovskite solar cells (PSCs) have emerged as an alternative option to silicon solar cells. PSCs belong to the third-generation technology of PV and have achieved remarkable breakthrough over the past decade,^6^ achieving an exceptional PCE of more than 25% and have the potential to outperform the Shockley Queisser limit.^7^

An incredible advantage PSCs is their compatibility with first- and second-generation solar cell technologies, opening the door to unlimited possibilities. Perovskite materials can be combined with conventional solar cells such as silicon and CIGS to create a cohesive tandem solar cells for exploring the untapped potential of high-performing PV cells.^8^ Furthermore, extensive research is ongoing to further enhance the performance of perovskite solar cells and their applications to further enhance technology.^9^

**Fig. 1:**
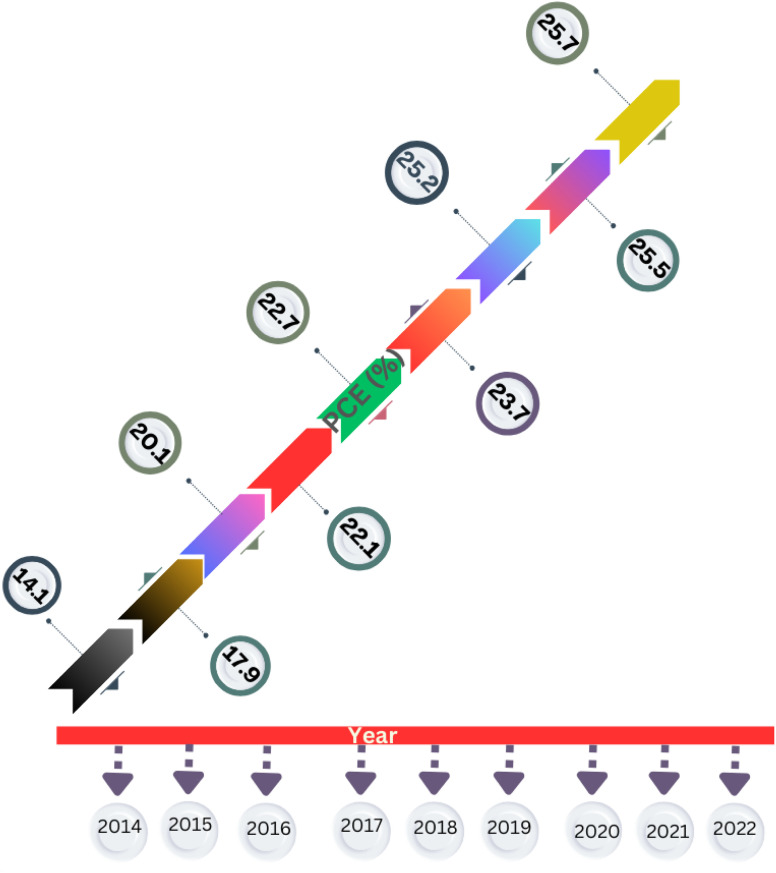
Progress in the PCE of PSCs in the last decade.

Kojima *et al.*^10,11^ carried out the ground-breaking work on the use of halide perovskites in solar cells. In 2009, these researchers achieved a PCE of up to 3.8% using methylammonium lead bromine (MAPbBr_3_) perovskite as a light sensitizer in dye-sensitized solar cells. This paved the way for future research in this area. The accomplishments achieved by perovskite materials during the last ten years are shown in Fig. 1, focusing on lead-based, tin-based, germanium-based, polymer-based, lead-free halide double perovskite-based and tandem PSCs.

For PSCs to thrive in the commercial landscape and effectively compete with established technologies, they must satisfy three critical requirements. Firstly, PSCs need to exhibit remarkable energy conversion efficiency, ensuring that they can generate power effectively. Secondly, these solar cells should demonstrate a prolonged operational lifespan, assuring users of their durability and long-term performance. Lastly, they need to be produced at low cost to enable the widespread implementation of low-cost PV technology. Achieving these requirements is essential to deliver power at an exceptionally competitive rate per watt, making PSCs a financially viable and attractive option for a broad range of applications. However, although PSCs have shown great promise, the current commercial systems still do not meet all these specifications completely. Accordingly, there is still a lot of room for further exploration in PSC technologies to achieve the full potential of highly efficient, durable, and cost-effective photovoltaic solutions.

Considering the pressing global need for sustainable energy solutions, the exploration and development of PSCs present a promising avenue towards achieving efficient, durable, and cost-effective photovoltaic technologies. This review presents a comprehensive analysis of the advancements and challenges in the field of PSCs, ranging from their foundational principles to the latest innovations in materials and design. By delving into the intricacies of the charge transport layers, the merits and demerits of lead and tin-based PSCs, and the pioneering work in germanium-based PSCs, we aim to provide a complete understanding of the current landscape. Furthermore, the exploration of A-site modification, the integration of polymer-based PSCs, the prospects of lead-free halide double perovskite solar cells, and the innovative tandem solar cells underscore the versatility and adaptability of PSCs. The journey of PSCs, from their inception to their current state, is a testament to the relentless pursuit of excellence in the renewable energy sector. As the global energy landscape evolves, it becomes increasingly evident that PSCs, with their promising attributes, hold significant potential to address the significant energy challenges presently. Accordingly, the aim of this review is to provide researchers, industry professionals, and enthusiasts with a consolidated resource, guiding future endeavors in the quest for sustainable and efficient photovoltaic solutions.

## Perovskite solar cells

**Fig. 2:**
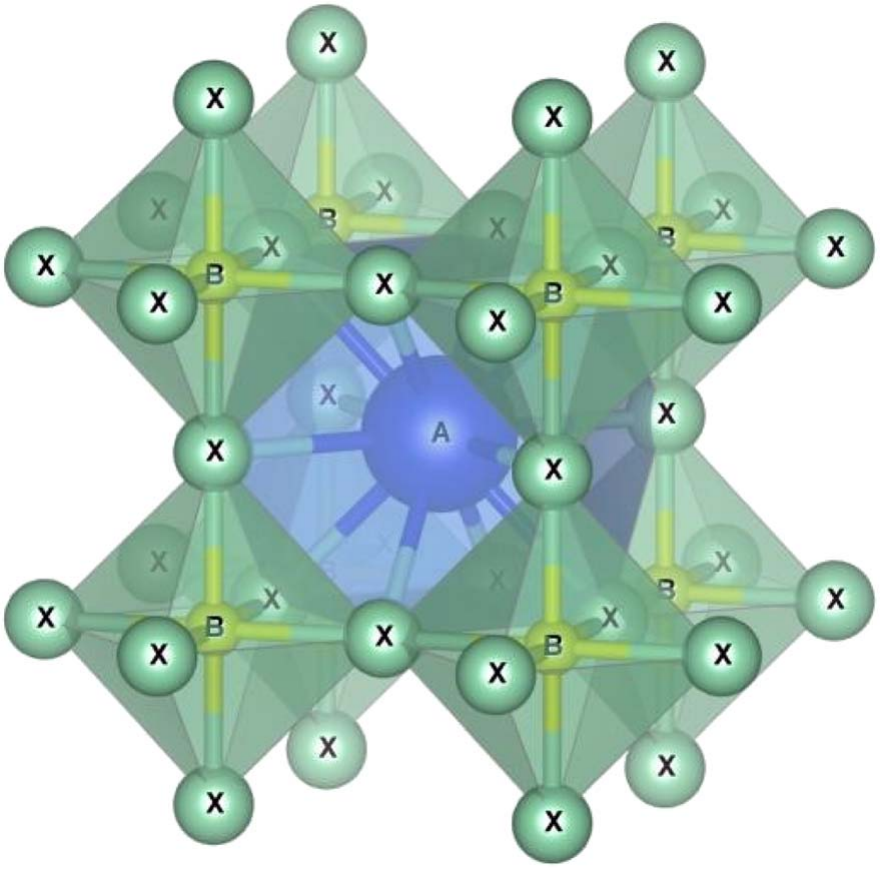
ABX_3_ crustal of perovskite materials.

**Fig. 3:**
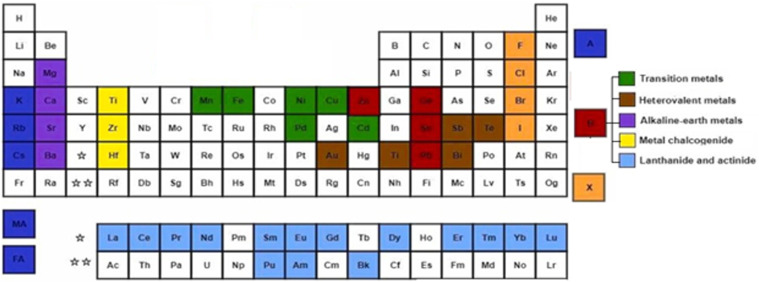
Elements used in perovskite ABX_3_.

Perovskites are materials possessing an ABX_3_ crystal structure, as shown in Fig. 2, where “A” stands for an organic/inorganic cation group, including cesium (Cs^+^), methylammonium (MA^+^, CH_3_NH^3+^), and formamidine (FA^+^, CH_3_CH_2_NH^3+^). Similarly, “B” stands for a metal cation group, such as Sn^2+^ and Pb^2+^. Alternatively, “X” is a halide, such as iodide, chloride and bromide. Based on this hybrid crystal structure, PCS have tunable band gaps (1.3–2.2 eV), high absorption co-efficient (5.7 × 10^4^ cm^−1^ at 600 nm), low Auger recombination, efficient carrier mobility (1–10 cm^2^ V^−1^ s^−1^) and long charge diffusion length.^12^Fig. 3 shows the elements that can be used in the ABX_3_ perovskite crystal structure.

The tunable bandgap of perovskite solar cells, which ranges from approximately 1.3–2.2 electron volts (eV), provides several distinct advantages. Firstly, it allows the optimization of their absorption in the solar spectrum. Given that the solar spectrum is broad, a tunable bandgap enables these cells to be specifically optimized for absorbing different parts of the spectrum, thereby enhancing the overall energy conversion efficiency. This feature is particularly beneficial in the development of tandem solar cells, where the perovskite layers with varying bandgaps can be stacked together or with other materials such as silicon to absorb different wavelengths, potentially surpassing the efficiency limits of traditional single-junction cells. Moreover, their tunable bandgap makes perovskite solar cells adaptable to various environmental conditions. For example, different geographic locations with unique solar irradiance profiles can benefit from cells optimized for specific conditions, increasing their efficiency in diverse climates.^13^ This adaptability is especially useful for maintaining higher efficiency in hotter climates, given that the efficiency of solar cells generally decreases with an increase in temperature, where a tunable bandgap can help mitigate this effect. Additionally, the versatility offered by the tunable bandgap extends to a range of applications. For instance, in building-integrated photovoltaics (BIPV), where aesthetic considerations are crucial, the ability to adjust the bandgap allows for different colorations and light absorption characteristics. This versatility also supports the use of thinner perovskite layers, reducing material costs and enabling the production of lighter and more flexible solar cells. In summary, the tunable bandgap of perovskite solar cells is a key attribute that significantly contributes to their efficiency, versatility, and potential for widespread application in various environments and integrations.^14^

Another critical attribute is their high absorption coefficient, which is notably around 5.7 × 10^4^ cm^−1^ at 600 nm. This high absorption coefficient signifies that perovskite materials can absorb a substantial amount of sunlight very efficiently, even when applied as thin films, which is particularly advantageous for several reasons. Firstly, it contributes to the high efficiency of perovskite solar cells. The ability to absorb a significant amount of light with a relatively thin layer of material means that more photons are converted into electrical energy, boosting the overall power conversion efficiency of the cell. This high efficiency is crucial for making solar energy a more viable and competitive source of renewable energy. Secondly, the high absorption coefficient enables the production of lightweight and flexible solar cells. Given that a thinner layer of material is required to achieve the desired absorption, perovskite solar cells can be made with less bulk, reducing their weight and allowing for more flexibility in their application.^15,16^ This aspect opens up new possibilities for the integration of solar cells, such as in portable and wearable electronics, unconventional building surfaces, and other areas were traditional, heavier solar panels may not be feasible. Furthermore, the efficient light absorption with a thinner thicknesses implies lower material usage, which can result in reduced manufacturing costs. This cost-effectiveness is essential for the widespread adoption and deployment of solar energy technologies, making renewable energy more accessible and affordable. Lastly, the high absorption efficiency at a specific wavelength such as 600 nm demonstrates the potential for perovskite solar cells to be finely tuned for specific parts of the solar spectrum, further enhancing their compatibility with multi-junction solar cell technologies. This compatibility can lead to the creation of highly efficient, multi-layered solar cells that can outperform traditional single-junction cells.

Continuing the discussion on the advantages of perovskite solar cells, the efficient carrier mobility of these materials, ranging from 1 to 10 cm^2^ V^−1^ s^−1^, stands out as a key attribute. This high carrier mobility is crucial in several aspects of the performance of solar cells. Firstly, it leads to improved charge collection. The rapid movement of charge carriers (electrons and holes) to the electrodes minimizes recombination losses, thereby enhancing the overall efficiency of the solar cell.^17^ In addition, this efficient carrier movement reduces internal resistive losses, ensuring that less energy is lost as heat and maintaining high efficiency during operation. This high carrier mobility is also beneficial under low-light conditions. It compensates for the lower number of photons, ensuring that solar cells maintain a good level of efficiency even when the light conditions are not ideal. Furthermore, the compatibility of high carrier mobility with thin film technology is particularly advantageous for perovskite solar cells. Despite the use of thin layers, the movement of charge carriers is not significantly hindered, which is vital for sustaining high efficiency in thin-film solar technologies.^18^ Another exciting aspect of the high carrier mobility in perovskites is its potential in applications beyond photovoltaics. For instance, in photodetectors and other optoelectronic devices, this property can lead to faster response times and higher sensitivities. Additionally, the high mobility in perovskites reduces the impact of impurities and defects, which typically hinder the performance in materials with lower carrier mobility. This means that even with minor imperfections, carriers in perovskites can still move effectively, reducing the need for ultra-high purity in material production.

The low exciton binding energy in perovskite materials is crucial for efficient solar energy conversion. In photovoltaic materials, excitons (bound pairs of electrons and holes) need to be separated into free charge carriers for the generation of electricity. The low exciton binding energy in perovskites means that less energy is required to separate these pairs, thereby facilitating efficient charge carrier generation under normal sunlight conditions. This leads to higher efficiency in converting solar energy into electrical energy. Moreover, the high dielectric constant of perovskite materials plays a pivotal role in enhancing their solar cell performance. A high dielectric constant leads to the efficient screening of charge carriers, reducing their recombination rate.^19^ This efficient photogeneration of electrons and holes ensures that more of the absorbed sunlight is converted into useable electrical energy, improving the overall power conversion efficiency of solar cells. In terms of practical solar cell metrics, these properties translate into significantly high short-circuit current densities and open-circuit voltages. The short-circuit current density indicates how much current the solar cell can produce under the optimal sunlight conditions, while the open-circuit voltage represents the maximum voltage the cell can generate when not connected to an external circuit. Both parameters are crucial for determining the overall efficiency of a solar cell, and perovskite materials excel in these aspects due to their inherent physical properties. Lastly, the long charge diffusion lengths in perovskite materials further contribute to their efficiency. The charge diffusion length is the average distance that charge carriers can travel before recombining. Longer diffusion lengths in perovskites imply that electrons and holes can travel farther, increasing the likelihood of their successful extraction and conversion into electrical energy. This characteristic is particularly beneficial in thin-film solar cells, where efficient charge transport across the material is essential for high performance. Table 1 shows the band gap, binding energy and carrier mobility of different perovskite materials.

**Table 1:** Band gap, carrier mobility, and exciton binding energy of different perovskite materials

S#	Perovskite materials	Band gap (eV)	Carrier mobility (cm^2^ V^−1^ s^−1^)	Exciton binding energy (meV)	References
1	MAPbI_3_	1.55 (tunable)	24.0 ± 6.8 (μe)	2–60	20–22
			105 ± 35 (μh)		
2	MAPbBr_3_	2.39–2.48	35 (μe, μh)	40–150	23–25
3	MASnI_3_	1.33 (tunable)	322 (μe)	29	26–28
			2320 (μh)		
4	CsPbBr_3_	2.0	63 (μe)	40	29–31
			49 (μh)		
5	CsPbI_3_	1.44	33 ± 5 (μe, μh)	20	29, 30 and 32
6	CsPbCl_3_	2.82	28 ± 1 (μe)	75	29, 30 and 33
			20 ± 1 (μh)		
7	FAPbI_3_	1.5	27 (μe, μh)	35	31, 34 and 35
8	FASnI_3_	1.27	103 (μe)	31	24, 28 and 34
			67 (μh)		

Presently, organic–inorganic hybrid perovskite (OHIP) materials have emerged as the optimal choice for cost-effective solar cell production with exceptional performance. Mitzi *et al.* first showed that an OHIP material could be used in light-emitting diodes and transistors in the 1990s.^36,37^ Moreover, OHIPs display distinct optical and electrical features in comparison to common organic and inorganic semiconductors. These OHIP materials also have a weak binding energy, large Bohr radius, high dielectric constant, long diffusion length and high carrier diffusion velocity, in addition to having an exceptional light absorption capacity.^38,39^ Owing to all these benefits, OHIP materials have risen to the top of the list of contenders for the fabrication of inexpensive, highly effective solar cells.

**Fig. 4:**
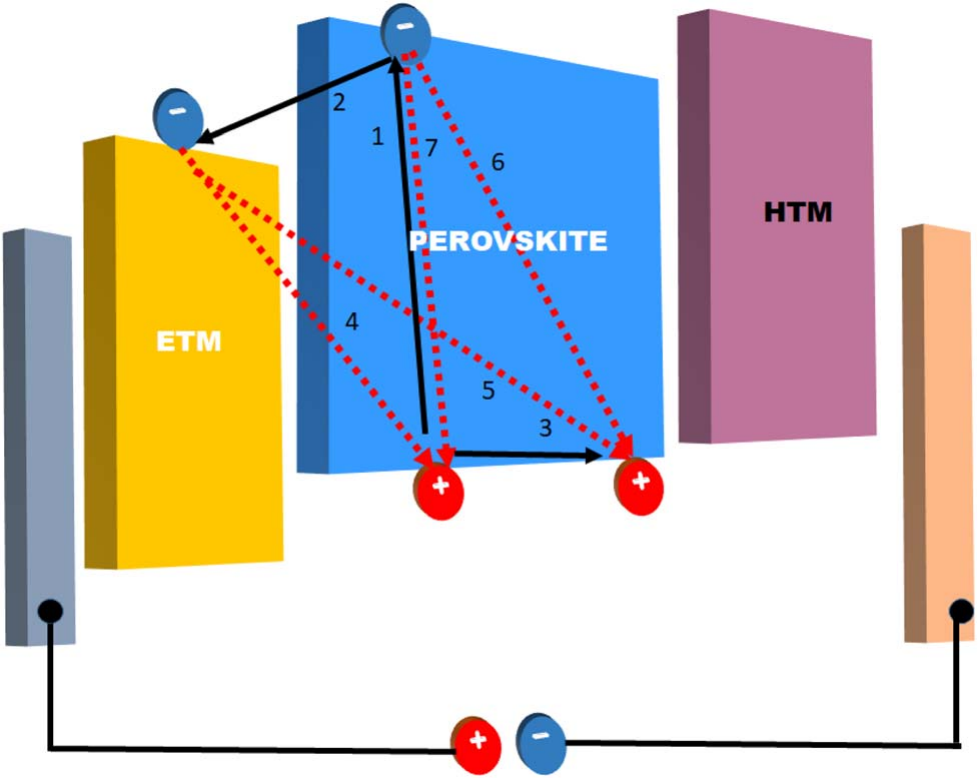
Energy levels with charge transfer processes inside a PSC.

In solar cells, perovskite materials such as CH_3_NH_3_PbX_3_ and CH_3_CH_2_NH^3+^SnX_3_ are used as the absorber. Additionally, an electron transport layer (ETL) and hole transport layer (HTL) are positioned on either side of the absorber. When exposed to light, the perovskite absorber introduces charge carriers (electrons – e^−^ and holes – h^+^), which are transported to the n-type and p-type charge transport layers (CTL), respectively, thereby generating free charge carriers. Subsequently, electrons migrate towards the cathode through the ETL and the external circuit. Simultaneously, the oxidized perovskite is regenerated and returns to its ground state with the assistance of the compact portion of the HTL. Consequently, the holes in the HTL diffuse in the opposite direction of the electrodes, where they recombine with the electrons, ultimately forming a current at the terminus of the circuit. Importantly, a relationship exists between the thickness of the perovskite material and the generation of a current.^40^Fig. 4 illustrates the energy levels involved in the charge transfer process in PSCs.

According to research, OHIP materials are particularly intriguing possibilities for solar cell applications. Additionally, atmospheric solution processing and easy preparation procedures such as vacuum deposition have been developed because of the abundant availability of the precursor components in OHIPs.^41,42^ Initially, OHIPs were used in solar cell applications by Miyasaka and colleagues by employing the CH_3_NH_3_PbX_3_ sensitizer in dye-sensitized solar cells (DSSC), with a PCE of 3.81%.^42^ With significant advancements in technology, the PCE has increased to as high as 25.8% within a short period.^43^

## Charge transport layers

The role of ETLs in the advancement of PSCs is important.^44^ ETLs consist of highly n-doped colloidal thin films designed to facilitate the transport of electrons from the perovskite layer to the cathode. The commonly employed ETL materials in PSCs include ZnO, TiO_2_, SnO_2_, and their mesoporous counterparts, which are traditionally used in conventional perovskite solar cells. However, oxide-based ETLs are characterized by broad grain boundaries and suffer from poor interface recombination.^45^ Notably, issues such as oxygen vacancies and trap-assisted recombination contribute to the emergence of defects in semiconductor ETLs. Thus, to address these limitations, researchers have explored alternative materials, including single-crystalline substances, to enhance the ETL performance in perovskite solar cells. This approach suggests that nanosheets or atomically thin forms of transition metal dichalcogenides such as WS_2_, TiS_2_, and MoS_2_ can serve as promising ETL candidates. Nanosheets are advantageous due to their one-atom-thick crystal structure, which minimizes defects.^46,47^ Moreover, their thin configuration facilitates rapid charge carrier transfer to the electrode.^48,49^

MoS_2_ is frequently chosen as the ETL due to its favorable attributes, including low trap density and robust carrier mobility.^50^ Malek *et al.* introduced a novel approach by demonstrating the direct production of MoS_2_ nanosheets on an indium-tin-oxide (ITO) substrate. Their investigation revealed the optimal homogeneity of the nanosheets at 200 °C. When these synthesized materials were employed as ETLs, they notably enhanced the interfacial charge transfer capabilities, stability, and overall performance of PSCs. It was observed that reducing the thickness of the MoS_2_ layer resulted in an increase in the PCE of the solar cell. Specifically, the thicker MoS_2_ nanosheet ETL exhibited a *V*_oc_ of 0.56 V, fill factor (FF) of 37%, *J*_SC_ of 16.24 mA cm^−2^, and PCE of 3.36%. Remarkably, even after continuous exposure to peak sunlight intensity for 80 s, these solar cells retained 90% of their initial PCE.^51^

Furthermore, owing to its ambipolar characteristics, MoS_2_ is sometimes employed as an HTL.^52^ An example of its versatility was demonstrated in the work by Kim *et al.* in 2016, where MoS_2_ was utilized as an HTL in a perovskite solar cell (PSC), resulting in an impressive PCE of 9.53%.^53^ Subsequently, Das *et al.* employed MoS_2_ as an HTL in an inverted p–i–n heterojunction planar PSC, achieving a PCE of 6.01%.^54^

In addition to ETLs, HTLs play a crucial role in optimizing the efficiency of solar systems. HTLs consist of highly p-doped materials designed to facilitate the movement of holes from the perovskite layer to the anode. Extensive research has been undertaken to enhance the conductivity of HTLs by doping with various substances, aiming to prevent charge carrier recombination at the HTL/perovskite interface.^55^ Among the HTL candidates, spiro-OMeTAD stands out due to its unique characteristics, including low glass transition temperature and good solubility. However, unprocessed spiro-OMeTAD exhibits a low PCE due to its insufficient oxidation states. In PSCs, their photovoltaic performance often relies on a prolonged oxidation process.^56^ Kim *et al.* addressed this challenge by expediting the oxidation of spiro-OMeTAD through exposure to oxygen plasma.^57^ Nevertheless, exposure to plasma can trigger the decomposition of the perovskite phase into PbI_2_. Thus, to circumvent this issue and enhance the hole-carrying capacity of spiro-OMeTAD, doping with trivalent (p-dopants) materials has been explored. This approach aims to simultaneously mitigate the decomposition of the perovskite and improve the hole transport in the HTL.

Presently, the utilized p-dopants range from metal–organic complexes to metal oxides and organic molecules.^58^ However, although these dopants exhibit potential benefits for PSCs, their limited solubility and intricate degradation processes hinder their widespread application. Thus, to address this challenge, innovative approaches have been explored. Cobalt (Co_60_) and FeCl_3_ complexes have shown promise as efficient p-type dopants by oxidizing spiro-OMeTAD, generating new holes and enhancing the conductivity.^59^ These dopants offer potential solutions to counteract the rapid aging of PSCs. Moreover, introducing an acid in the system can expedite the oxidation process and extend the operational life of these solar cells.^60^ Recent research efforts have focused on enhancing HTLs by incorporating acids in spiro-OMeTAD. In the study by Guan *et al.*, they investigated the impact of benzoic acid on the oxidation of spiro-OMeTAD, building on prior findings.^61^ Their results indicated that increasing the concentration of benzoic acid accelerated the oxidation and improved the hole-transmitting properties of the HTL. Moreover, optimizing the doping concentration effectively reduced the hysteresis in PSCs based on the HTL, resulting in an enhanced PCE of 16.26% under conventional AM 1.5 G illumination.

Yang *et al.*^62^ created a fluorinated spiro-OMeTAD for solar cell applications that was inexpensive and devoid of dopants. They claimed that the material was dopant-free and sensitive enough to be used as the HTL in CsPbI_2_Br-based PSCs. Next, they used 2,3,5,6-tetrafluoro-7,7,8,8-tetracyanoquinodimethane (C_12_F_4_N_4_) to modify the surfaces of CsPbI_2_Br perovskite and fluorinated spiro-OMeTAD. In comparison to the doped PSCs, the modified and dopant-free CsPbI_2_Br perovskite solar cells exhibited a very high PCE of 14.42% with a high *V*_OC_ of 1.23 V. Even after 30 days of open-air aging without encapsulation, the solar cells manufactured from the recommended HTL materials maintained 94% of their initial PCE, demonstrating exceptional longevity. Thus, high-performance CsPbI_2_Br PSCs can be fabricated using the produced dopant-free HTL. As a new technique for solution processing, a novel solvent mixture consisting of organic amines (H_2_O/ETA/EDA/DTA) in the volumetric ratio 2 : 6 : 1 : 1 was introduced by Zhao *et al.*^63^ to produce CuSeCN thin films for HTL application in p–i–n PSCs. For the developed HTL-based PSCs, they reported a PCE of 15.61% in the forward IV analysis and 15.97% in the reverse IV analysis. Importantly, these CuSeCN-based PSCs demonstrated minimal hysteresis and exceptional long-term stability. These results highlight the significant potential of CuSeCN films as HTL materials in solar applications.

Another ETL, ZnO, has attracted attention as a promising candidate for solar cell applications, owing to its high electron mobility, diverse nanostructured morphologies, and versatile growth methods.^64,65^ It is well-established that the efficiency of PSCs depends on the surface morphology and crystalline characteristics of the perovskite top layer. In this case, the morphology of the active perovskite layer, including surface roughness and particle size, is influenced by the choice of solvent during its creation, which can significantly impact the solar cell performance.

Recently, Ahmadi *et al.* introduced an economical method involving an ultrasonic bath to produce ETLs for perovskite solar cells using ZnO nanoparticles synthesized in three different solvents including isopropyl alcohol (IPA), 2-methoxy ethanol (2 ME), and ethanol.^66^ The research findings on the structure, morphology, and device performance revealed that the ZnO layers produced using 2 ME as the solvent exhibited the highest quality. Notably, a PSC utilizing ZnO (2 ME) as the electron transport layer and methylammonium lead iodide (MAPbI_3_) as the perovskite layer achieved an impressive power conversion efficiency (PCE) of 22%. This superior performance was attributed to the excellent MAPbI_3_ surface coverage, larger grain sizes, and the lowest defect density at the ETL/MAPbI_3_ interface. Consequently, ZnO-ETL-based solar cells emerge as a compelling choice for various solar cell applications.

Furthermore, due to its exceptional optical transparency, ZnSnO (ZTO) holds promise as an ETL material for solar cells. The oxygen vacancies naturally present in ZTO significantly facilitate charge carrier transmission. However, the presence of multiple oxygen vacancies in ZTO is considered a major drawback. Thus, Miao *et al.* addressed this issue by fabricating solar cells using ZTO doped with varying concentrations of silicon to investigate the impact of oxygen vacancies in ZTO and strategies for their management.^67^ They achieved this by producing amorphous metal oxide films through RF magnetron sputtering and adjusting the silicon concentration. Their research revealed a reduction in oxygen vacancies relative to silicon content, which was corroborated by X-ray photoelectron spectroscopy (XPS) analysis. Notably, the decrease in oxygen vacancies in silicon-doped ZTO (SZTO) contributed to improved charge extraction and conduction capabilities. Utilizing synthesized the SZTO as the ETL, they developed a PSC with impressive performance metrics, including a peak PCE of 13.4%, *J*_SC_ of 21.6 mA cm^−2^, FF of 0.67%, and *V*_OC_ of 1.04 V.

TiO_2_-based solar cells have achieved remarkable PCEs exceeding 20%. However, they have certain drawbacks. Thus, the choice of TiO_2_ as the ETL significantly influences device performance in all of the aforementioned methods. When utilized as an ETL in n–i–p structured PSCs on exposure to UV light, TiO_2_ can exhibit a rapid drop in *J*_sc_ and make the cells unstable.^68^ Thus, to address these limitations and protect PSCs from UV-induced degradation, several studies have explored the use of an interfacial layer positioned between the perovskite layer and the TiO_2_ ETL.^69^

Consequently, it is crucial to develop stable and high-performance PSCs, which has attracted substantial attention in this research direction.^70^ The stability of various ETL materials under ultraviolet (UV) radiation has driven this interest. Mg_*X*_Zn_1−*X*_O (MZO), due to its robust electron mobility and deeper conduction band, has emerged as a promising ETL material for PSCs.^71^ Accordingly, Han *et al.* recently demonstrated the exceptional stability of PSCs utilizing MZO-based ETLs when exposed to UV light.^72^ They highlighted that MZO exhibits enhanced carrier mobility and a more efficient conduction mechanism compared to TiO_2_. This characteristic prevents charge accumulation at the perovskite/MZO interface and facilitates efficient charge conduction between the two materials. The researchers achieved an MZO-based device with an impressive *V*_oc_ of 1.11 V and efficiency of 19.57%.

Notably, the MZO-based device retained 76% of its original *J*_sc_ compared to the TiO_2_-based device, which retained only 12% of its initial *J*_sc_. Both cells were analyzed following a year of aging under ambient conditions, including 40% to 80% relative humidity (RH) and 8 h of UV exposure. This exceptional UV resistance was attributed to the reduced electron capture site density in the MZO-ETL when exposed to UV light. The oxygen vacancies and zinc interstitials in MZO-ETL played a pivotal role in the ability of this material to withstand UV radiation without compromising the integrity of the perovskite active layer. Therefore, MZO as an ETL holds promise for the fabrication of durable PSCs resilient to UV light-induced degradation.

**Fig. 5:**
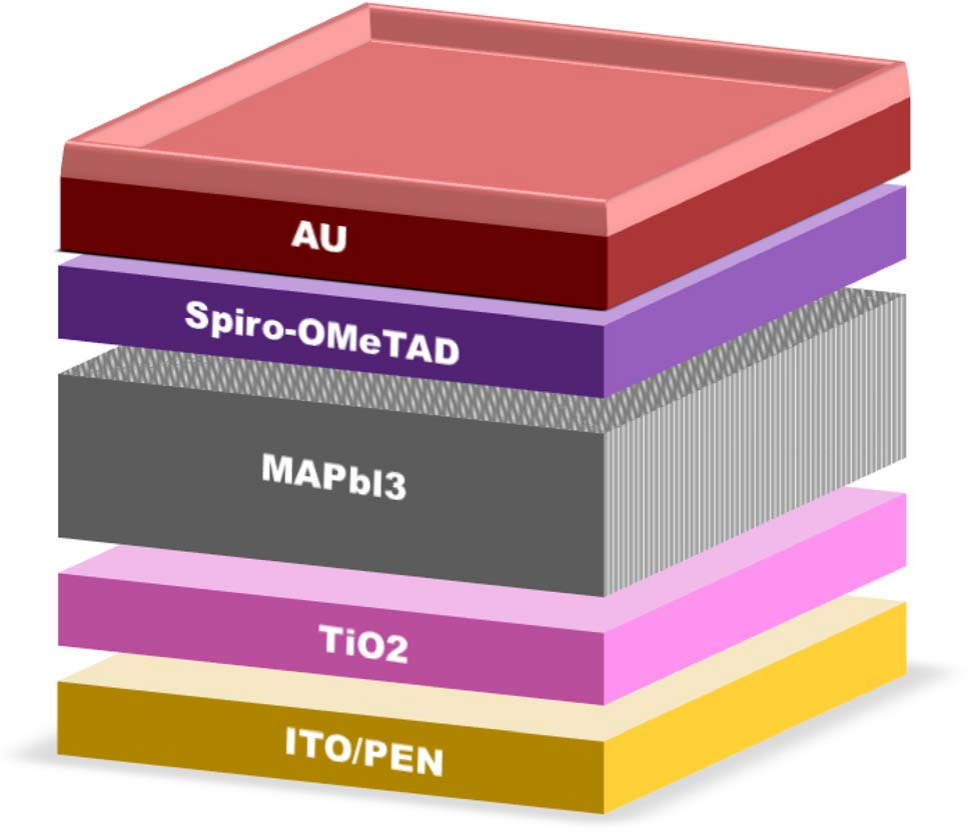
Architecture of c-TiO_2_-based PSCs.

Teimouri *et al.*^73^ demonstrated that lithium (Li) doping of TiO_2_ enhances the conductivity and electron transport in the ETL. Ultrasonication was employed to fabricate Li-doped TiO_2_ films, which exhibited improved conductivity and reduced solar power loss compared to undoped TiO_2_. Simulations conducted using a solar cell capacitance simulator (SCAPS) unveiled the impact of varying Li concentrations on the efficiency of perovskite solar cells. These simulations demonstrated a notable enhancement in performance, with the Li-doped electron transport layer (ETL) achieving a significantly higher PCE of 24.23% compared to the undoped ETL, marking an impressive increase of 1.97%. Additionally, Li-doped TiO_2_ showed a lower trap density between the absorber and ETL. These findings establish Li-doped TiO_2_ as a strong contender as the ETL in PSCs. In another study, Yang *et al.* employed a sol–gel production process with varying TiO_2_ concentrations to fabricate compact TiO_2_ ETLs (c-TiO_2_).^74^ Among them, the ETL prepared with the highest TiO_2_ concentration of 2.0 M exhibited the most noteworthy characteristics. The PSCs utilizing c-TiO_2_ achieved an impressive PCE of 16.11% and high *V*_oc_ of 1.1 V. This innovative use of c-TiO_2_ in PSCs represents a promising avenue for enhancing the efficiency of low-temperature solar panels. Fig. 5 provides a schematic representation of PSCs incorporating c-TiO_2_.

In another study, Zhang *et al.*^75^ employed a straightforward sintering technique to fabricate MgTiO_3_-coated TiO_2_ mesoporous scaffold layers with diverse treatment concentrations, targeting their application in solar cells. The photovoltaic performance was much improved once the manufactured scaffolds were used as shell layers. Furthermore, the MgTiO_3_ shell effectively blocked charge carriers from recombining at the MAPbI_3_/TiO_2_ interface. The crystallinity of MAPbI_3_ was improved by the incorporation of MgTiO_3_, which was crucial for the manufacturing of excellent-quality perovskite films. The PSC treated with an optimal concentration of 0.10 M achieved a remarkable PCE of 10.39%. Even after 1008 h of exposure to normal humidity, the device retained 88.35% of its initial PCE. The durable, highly-efficient MgTiO_3_-coated TiO_2_ mesoporous scaffold layers are promising materials for future solar systems, given their ease of manufacturing and exceptional performance.

The high electron mobility, good chemical stability, anti-reflectiveness and wide bandgap of indium oxide (In_2_O_3_) thin films make them a promising ETL material in PSCs.^76^ However, the hygroscopic nature of In^3+^ causes pinholes, fractures, and unfavorable morphology, as demonstrated in prior research due to the reaction between In^3+^ cations and water molecules during the fabrication of the samples.^77^ This hinders the potential of In_2_O_3_ as an ETL frontrunner in the solar cell industry. Thus, to enhance the PCE of photovoltaic devices, it is preferable to manufacture In_2_O_3_ free of flaws.

In the study by Zhang *et al.*, they pioneered the synthesis of stable In_2_O_3_ films at low temperatures using an exceptionally stable indium precursor solution.^78^ These films were intended for use as reliable ETL in PSCs. With a water content of only 0.2%, the indium precursor exhibited exceptional stability in ethanol. Adding the chelate ligand acetylacetone (acacH) to the solution prevented the further hydrolysis of the indium.^78^ All these factors contributed to the production of an In_2_O_3_ layer under the conditions of around 200 °C and relative humidity of 40–50%. The fabricated In_2_O_3_-film significantly enhanced the electron extraction and charge transfer at the ETL-perovskite interface. Air-processed PSCs with a compact In_2_O_3_ film achieved an impressive PCE of 13.97%, surpassing the PCE of 9.81% for the pure In_2_O_3_ film. These indium-based PSCs demonstrated long-term stability, retaining 94% of their PCE after 31 days of storage. Remarkably, utilizing In_2_O_3_ films as the ETL greatly enhances both the stability and PCE in air-processed PSCs.

In a distinctive strategy aimed at boosting the PCE of PSCs, Tseng *et al.* proposed improvements in the charge concentration, interface quality, and morphology of the Cu_2_O/MAPbI_3_/SiO_2_ hetero-structure by utilizing RF magnetron sputtering.^79^ They successfully fabricated an ultrathin SiO_2_ ETL and Cu_2_O HTL. The incorporation of Cu_2_O as the HTL and SiO_2_ as the ETL resulted in an enhanced PCE, reduced charge carrier recombination and remarkable suppression of pinholes and defects. The device achieved a peak PCE of 18.4% due to the improved *V*_OC_. This highlights the potential of various inorganic materials, including Cu_2_O and SiO_2_, as promising candidates for PSCs.

Recently, high-performance PSCs were fabricated by Zhu *et al.* by introducing a thin m-TiO_2_ layer at the interface between a perovskite film and compact TiO_2_ in a planar PSC.^80^ This interfacial modifying layer boosted the hardness and particle size of the perovskite films. Solar cells incorporating this layer achieved a superior performance, with an impressive PCE of 18.5% and a low hysteresis coefficient of 4.5%, surpassing conventional planar and mesoporous cells. The interfacial modifying layer holds promise for next-generation PSCs, enabling high transport capacity and improved carrier separation efficiency.

Hu *et al.* introduced a distinctive approach known as the multifunctional interface layer (MFIL) technique to enhance the PCE in inverted PSCs.^81^ MFIL serves multiple purposes, including trap passivation, electron transport, ion migration suppression, moisture barrier, and near-infrared photocurrent enhancement. This multifunctional approach contributes to the enhanced efficiency and durability of devices. When considering environmental conditions such as heat, moisture, and light, the MFIL-fabricated device showed outstanding stability for up to 1700 h without encapsulation and a considerable PCE of 21%. Molecular orientation studies at the perovskite/MFIL interface have shed light on maximizing the device performance by increasing the molecular bonding at the interface and decreasing the trap density *via* the design of sophisticated interlayers.

The suggested MFIL method offers a new chance to boost the efficiency of future perovskite-based photovoltaic systems, as shown by the findings of the above-mentioned study. For perovskite solar cell applications, Yun *et al.*^82^ showed the production of well-ordered ZnO nanorods on an FTO substrate using a low-temperature water bath method. These nanorods exhibited varying lengths depending on the reaction time and offered advantages such as improved electron (e^−^) transportation, increased contact area, high visual transmittance, and a compact interface. By utilizing these ZnO nanorods as the ETL layer in solar cells, an impressive PCE of 14.22% was achieved under AM 1.5 G illumination. This highlights the potential of ZnO-based materials as effective ETLs in high-performance PSCs.

**Fig. 6:**
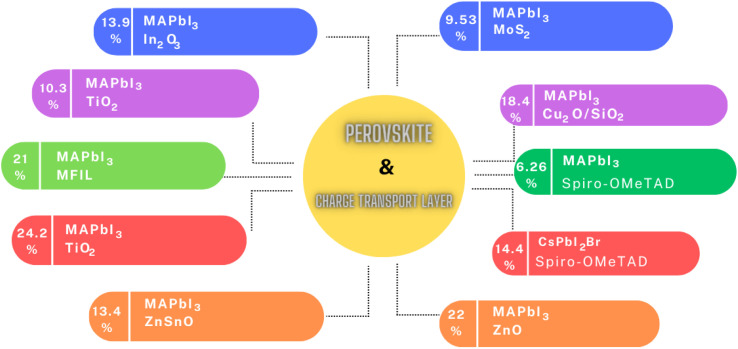
Effect of different CTLs on the performance of PSCs.

In summary, the intricate landscape of ETLs and HTLs in PSCs has been thoroughly examined, revealing their pivotal role in optimizing the efficiency and stability of these solar systems, as shown in Fig. 6. From traditional oxide-based ETLs to innovative materials such as nanosheets, MoS_2_, and fluorinated spiro-OMeTAD, the advancements in this domain underscore the relentless pursuit of achieving higher PCEs. The exploration of various doping techniques, the introduction of multifunctional interface layers, and the utilization of novel materials such as ZnO nanorods further emphasize the in-depth research and innovation in this field. Table 2 shows the summary of the different CTLs and their impact on the performance of PSCs discussed above. As the global demand for sustainable energy solutions continues to grow, the findings presented in this section highlight the potential of PSCs to revolutionize the solar industry. The continuous endeavors to address the challenges, enhance the performance, and ensure the long-term stability of PSCs pave the way for a brighter, more sustainable future.

**Table 2:** Progress in charge transport layer materials for PSCs

Perovskite	CTL (charge transport layer)	Methodology	Significance/improvements	References
MAPbI_3_	MoS_2_	Direct production of MoS_2_ nanosheets on ITO substrate	Enhanced interfacial charge transfer capabilities, stability, and overall performance	50
MAPbI_3_	MoS_2_	Used as HTL	Versatility of MoS_2_ as both ETL and HTL	52
MAPbI_3_	MoS_2_	Inverted p–i–n heterojunction planar PSC	Utilization of MoS_2_ as HTL	54
MAPbI_3_	Spiro-OMeTAD	Incorporation of benzoic acid	Accelerated oxidation and improved hole-transmitting properties	61
CsPbI_2_Br	Spiro-OMeTAD	Fluorinated spiro-OMeTAD without dopants	High PCE and exceptional longevity	62
MAPbI_3_	ZnO	Ultrasonic bath to produce ZnO nanoparticles in different solvents	Superior performance due to excellent MAPbI_3_ surface coverage and low defect density	66
MAPbI_3_	ZnSnO	Doping with silicon	Improved charge extraction and conduction capabilities	67
MAPbI_3_	TiO_2_	Lithium (Li) doping of TiO_2_	Enhanced conductivity and reduced solar power loss	73
MAPbI_3_	TiO_2_	Sol–gel production process with varying TiO_2_ concentrations	Enhanced efficiency of low-temperature solar panels	74
MAPbI_3_	TiO_2_	MgTiO_3_-coated TiO_2_ mesoporous scaffold layers	Improved photovoltaic performance and durability	75
MAPbI_3_	In_2_O_3_	Synthesis of stable In_2_O_3_ films at low temperatures	Enhanced electron extraction and charge transfer	78
MAPbI_3_	Cu_2_O/SiO_2_	RF magnetron sputtering	Enhanced PCE, reduced charge carrier recombination	79
MAPbI_3_	TiO_2_	Introduction of a thin m-TiO_2_ layer at the interface	Superior performance and low hysteresis coefficient	80
MAPbI_3_	MFIL	Multifunctional interface layer (MFIL) technique	Enhanced efficiency and durability	81
MAPbI_3_	ZnO	Production of well-ordered ZnO nanorods on FTO substrate	Improved electron transportation and increased contact area	82

## Lead-based PSCs

Kojima *et al.* employed MAPbBr_3_ and MAPbI_3_ as the first perovskite materials used in DSSCs as sensitizers.^42^ Since then, Pb-halide-based perovskites have drastically transformed the field of PVs. The photocurrent and photovoltage of the device are significantly influenced by the inclusion of I and Br ions as halide anions in PSCs. Pb-based perovskites possess several exceptional qualities, making them suitable for optoelectronic applications. They are characterized as direct bandgap (1.6 eV) semiconductor materials with a Shockley–Queisser limit for single-junction solar cells that is nearly ideal (1.43 eV). They exhibit an exceptionally high absorption coefficient (5 × 10^4^ cm^−1^), which surpasses that of GaAs and is nearly 25 times greater than that of Si.^83^ The balanced effective masses of electrons (e^−^) and holes (h^+^) facilitate enhanced charge carrier transport.^84^ In addition, photo-generated charge carriers have a long lifespan, complemented by a 1 m diffusion length.^85^ There are two architectural designs for perovskite single-crystal devices, *i.e.*, planar and lateral structures. Between them, the more commonly used solar cell design is the planar or conventional sandwiched structure. In the work by Malinkiewicz *et al.*, they employed MAPbI_3_ (a donor material paired with fullerene derivatives) to fabricate a planar-structured single-crystal device. Their planar devices were constructed at room temperature using a thin 285 nm MAPbI_3_ coating, thermally positioned between materials that acted as electron acceptors (PCBM) and electron blockers (polyTPD).^86^ The resulting device achieved a pioneering PCE of 12%, setting a benchmark for fullerene-based organic solar cells. Subsequently, small molecule-based hybrid solar cells have reported PCEs exceeding 18%.^87^ However, planar-structured devices typically maintain a planar design, where lateral-structured single-crystal devices offer enhanced mechanical and thermal properties, endowing the device with increased stability.

Lateral structures do not require costly substrates such as ITO and are free-standing.^88^ This immediately influences their capacity to absorb light given that they can avoid being absorbed by the glass substrate and conductive electrode, which improves the photocurrent and efficiency in comparison to conventional PSCs. Thus, as integrated back contact structures, lateral structures are more effective and produce devices that are less expensive. By simply improving the anode contact, Y. Song *et al.* created a stable and effective single-crystal MAPbI_3_ lateral structure that was surface treated with MAI and resulted in improved *V*_OC_ and FF as well as increased PCE reaching 11%.^89^ The fundamental challenge in producing this type of building on a large scale is the use of challenging photolithography and laborious deposition procedures. Although MAPbI_3_ is an effective material, due to its primary drawback of low stability, researchers have attempted to tailor this material by adjusting its composition *via* the synthesis of chloride or bromide counterparts (*e.g.*, MAPbI_3−*x*_Cl_*x*_ and MAPbBr_3_, respectively).^90,91^ For example, compared to iodide-based perovskite solar cells (MAPbI_3_, 1.15 V), the *V*_OC_ jumped to 1.3 V in MAPbBr_3_.^92^

The optoelectronic characteristics of polycrystalline organic inorganic hybrid perovskite (OIHP) thin films may be negatively impacted by the excess charge traps at their grain boundaries.^93^ Furthermore, polycrystalline perovskites are known to be susceptible to moisture and photodegradation. A single crystal OIHP, such as MAPbX_3_, usually has structural characteristics such as crack-free, smooth surfaces and well-shaped boundaries, and they are also thermally more lasting than their polycrystalline counterparts.^94^ Additionally, single crystals have diffusion lengths and carrier mobilities that are around two orders of magnitude greater than that of their polycrystalline phases.^95^ These improved characteristics cause the PCE of single-crystal PSCs to quickly increase, rising from 6.53% to 22.8% during the last three years.^96^ Consequently, single-crystal perovskites are excellent candidates for the fabrication of solar cells that are both stable and effective when used in the development of industrial applications. However, single-crystal perovskites are associated with some drawbacks, such as the inability to synthesize or develop large-area thin films with excellent quality.

The presence of Pb in the perovskite crystal is a significant disadvantage despite the high PCE of MAPbX_3_ perovskite materials. Exposure to Pb is very poisonous and bad for the health. Humans who consume it may have hyperactivity and neurological, reproductive, and renal organ damage.^97^ Thus, researchers have focused on lowering the content of lead or removing it in PSCs given that these toxicity concerns restrict the use of Pb-containing PSCs.^98^ Another strategy to reduce the total Pb consumption in the device, while keeping the high PCE, Zheng *et al.*^99^ recommended the physical Pb reduction idea. Perovskite technology is now making gradual progress toward Pb-free materials by giving priority to research on workable substitutes.

Table 3 presents a summary of the recent development in Pb-based PSCs. The advancement in Pb-halide-based perovskites has undeniably ushered in a transformative era in PVs, with their remarkable optoelectronic properties setting new benchmarks in solar cell efficiencies. From the pioneering work of Kojima *et al.* to the innovative approaches by Malinkiewicz and Song, the versatility and potential of these materials have been consistently demonstrated. However, the journey of Pb-based PSCs also has challenges. The inherent instability of MAPbI_3_ and the detrimental effects of the grain boundaries in polycrystalline structures have driven researchers towards the exploration of single-crystal perovskites, which promise enhanced stability and performance. However, the overarching concern remains the toxic nature of lead, which poses significant environmental and health risks. The endeavors of researchers to mitigate the lead content in PSCs underscore the commitment of this industry to developing sustainable and safe perovskite technologies. As the field progresses, the quest for efficient, stable, and lead-free perovskite materials will undoubtedly remain at the forefront, guiding the future trajectory of perovskite solar cell research and applications.

**Table 3:** Progress in lead-based PSCs

Perovskite type	Methodology	Significance/improvements	References
MAPbBr_3_ and MAPbI_3_	First used in DSSCs as sensitizers	Pioneered the use of perovskite materials in DSSCs	36
Pb-halide-based perovskites	Incorporation of I and Br ions	Transformed the field of PVs; influenced photocurrent and photovoltage	36
Pb-based perovskites	Direct bandgap (1.6 eV) semiconductor	Nearly ideal Shockley–Queisser limit for single-junction solar cells (1.43 eV)	76
MAPbI_3_	Planar structured single crystal device with fullerene derivatives	Achieved a PCE of 12%	79
MAPbI_3_ lateral structure	Surface treated with MAI	Improved *V*_oc_, FF, and achieved a PCE of 11%	82
MAPbBr_3_	Bromide-based perovskite	Higher *V*_oc_ compared to iodide-based perovskite solar cells	85
Single crystal OIHP	Superior characteristics compared to polycrystalline	Rapid increase in PCE from 6.53% to 22.8%	89
Sn and Ge mixtures	Reduction of Pb concentrations	Predicted decrease in toxicity	91
Physical Pb reduction	Strategy to use less Pb in the device	Maintain high PCE while reducing Pb consumption	92

## Tin (Sn)-based PSCs

Lead (Pb) is commonly present in PSCs. However, although lead-based PSCs exhibit an impressive perform, the toxicity of Pb poses significant environmental concerns and hinders the mass production and commercialization of PSCs. Thus, to make PSCs more affordable and environmentally friendly, it is necessary to develop Pb-free or Pb-reduced perovskites.^100,101^ When considering replacements for the hazardous Pb in perovskite unit cells, factors such as the ionic radius and stability of the perovskite structure are vital. In this case, divalent metallic ions such as strontium (Sr), tin (Sn), calcium (Ca), and barium (Ba) have emerged as potential candidates to replace Pb, aligning with Goldschmidt's octahedral factor and tolerance principles.^102,103^ Among them, Sn has attracted significant attention due to its electron configuration and coordination geometry, which are similar to that of Pb. Generally, Sn-based perovskites, represented by the chemical formula ASnX_3_, are considered the best alternatives to Pb. Sn-based PSCs boast attributes such as low excitation binding energies, superior carrier mobility, and a theoretical PCE of 33%, making them favorable compared to Pb-based devices. However, their efficiency of 10% is notably lower than that of Pb-based perovskite solar cells. Moreover, the oxidation of Sn^2+^ to Sn^4+^ adversely affects the stability of PSCs.^104^ The partial replacement of divalent metal-ions for lead may enhance the performance of perovskite solar cells, while posing no environmental hazards.

Considering with this, Ji *et al.*^105^ fabricated a PSC with a Pb–Sn mixed triple cation, which displayed a PCE of 16.10%. Bulky organic ligands are also included, which affect the orientation and development of the grains, causing an increase in spin orbit coupling (SOC) and out-of-plane photoinduced bulk polarization. These factors determine how well 2D/3D perovskite solar cells function photovoltaically. The SOC specifically increases the photovoltaic activity, *i.e.*, a higher SOC greatly enhances the spin conversion from optically generated states. In contrast to spin-forbidden recombination, which creates dark states, spin-allowed recombination creates brilliant states. Alternatively, the vertically oriented grains in 2D/3D perovskites with condensed traps confer benefits in terms of aligning optical transition dipoles, thereby amplifying the photovoltaic performance. Recently, Zhang *et al.*^106^ fabricated significantly proficient PSCs by introducing PEA^+^ (bulky organic cations) in 2D/3D Pb–Sn alloys using the solvent optimization approach. The growth-oriented orientations and SOC in Pb–Sn perovskites are modulated by organic cations after inclusion, which results in photoinduced bulk-polarization. The photovoltaic activities are improved by the increased SOC and bulk polarization. They stated that a high PCE of 15.93% is present in the 2D and 3D Pb–Sn alloy-based PSCs.

A good device performance in PSCs is facilitated by a suitable ETL.^107^ In this case, metal oxides have attracted significant interest in the development of ETL materials for solar cell applications due to their intrinsic characteristics, including robust thermal and chemical durability, elevated permittivity, and superior electrical conductivity.^108^ Presently, TiO_2_ is the most common material used to create very effective PSCs.^109^ However, it has certain drawbacks, including limited electron mobility (0.1–1 cm^2^ V^−1^ s^−1^), the need for high sintering temperatures (>450 °C), and perovskite deterioration in the presence of light.^110^ Therefore, researchers are focused on the introduction of alternative ETL materials to avoid these problems. Binary metal oxides such as ZnO and SnO_2_ are considered to be feasible replacements for TiO_2_ because of their improved electron mobility and convenient low temperature production.^111,112^ Nevertheless, ZnO-based photovoltaic setups suffer from inadequate stability arising from residual OH on the surface of ZnO, leading to the degradation of the perovskite structures. Hence, SnO_2_ has recently become popular as an ETL for PSCs. It has been shown that superior-quality SnO_2_-based devices have demonstrated excellent PCEs comparable to TiO_2_-based devices. Additionally, SnO_2_-based devices are more reliable than TiO_2_- and ZnO-based ones.^113^ Presently, significant research is devoted to altering the structure of ETL materials to increase the durability and efficiency of PSCs.

Generally, the oxidation characteristic of Sn has a significant impact on the operation of the device by generating vacancies inside cells. Thus, to avoid this, Mohammadian *et al.*^114^ showed how to make inexpensive, environmentally friendly tin-based PSCs without an HTL by using a natural antioxidant, *i.e.*, uric acid (UA). They claimed that the performance of the device is enhanced by the addition of UA because it lowers oxidation and promotes carrier recombination. This indicates that uric acid enhances the operation of the device by stopping the oxidation of Sn. Additionally, Ghahremani *et al.*^115^ employed strong pulsed light for the first time to quickly anneal the ETL (SnO_2_) and triple cation perovskites with the aim to fabricate effective PSCs. The inclusion of di-iodomethane alkyl-halide (CH_2_I_2_) prevented the regular crystallization during intensive pulsed light annealing and enhanced the surface characteristics of the perovskite layer by delivering iodine slices *via* UV radiation. They reported that the greatest efficiency of 12.56% was achieved by the SnO_2_-based PSC produced by intensive pulsed light annealing. Additionally, SnO_2_ quantum dots were used as the ETL in the creation of exceptionally proficient PSCs by Vijayaraghavan *et al.*^116^ They created SnO_2_ quantum dots using the low-temperature solution processing approach. The electron extraction and hole-blocking capabilities of SnO_2_-quantum dots were better than that of high-temperature-produced ETLs. Additionally, the device created employing SnO_2_-quantum dots as the ETL exhibited a high PCE of 13.64%.

In another study, Deng *et al.*^117^ presented a novel method for the preparation of tin-doped TiO_2_ ETL materials for PSC applications. To passivate the TiO_2_ film and surface, while doping TiO_2_ with Sn, they utilized hydriodic acid (HI) for the first time. Initially, HI regulates the hydrolysis of TiO_2_ and eliminates the trap centers of associated oxygen vacancies. Subsequently, TiO_2_/SnO_2_ films are created by incorporating Sn in HI-passivated TiO_2_. The use of SnO_2_ significantly improves the electron mobility, while suppressing the flaws over the whole film. Additionally, they reported that the 0.05 M SnO_2_-doped TiO_2_-based perovskite device showed low hysteresis, outstanding stability, and an efficiency of 17.77% among the generated samples. Additionally, the TiO_2_/SnO_2_ (0.05 M) device maintained 86% of its original efficiency even after sustained heating at 100 °C for 21 h. Therefore, compared to pristine TiO_2_-based devices, the inclusion of SnO_2_ improved the stability and efficiency of perovskite solar cells. To enhance the electron coupling, passivate trapping defects, and align the energy levels optimally at the junction of the perovskite and ETL layer, Zhang *et al.*^118^ implemented compact and ultra-thin SnO_*x*_ coatings produced from SnCl_4_. Furthermore, the PCE of the PSC based on Cl–SnO_2_ and SnO_2_ as the ETL was 18.6%, while that of the same device without Cl was 16.3%. Additionally, Huang *et al.*^119^ reported the addition of LiCl to the SnO_2_ ETL using a straightforward low-temperature procedure. The conductivity of SnO_2_ was greatly increased by the addition of LiCl, which enhanced the charge transport and prevented charge recombination. They reported that although the same device exhibited a PCE of 18.35% in the steady state, the PCE of the PSC based on Li:SnO_2_-ETL reached 19%.

**Fig. 7:**
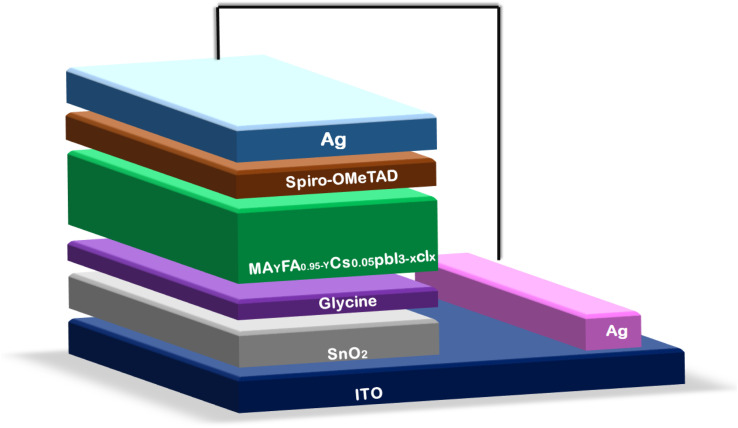
Schematic of the device architecture of an SnO_2_-based PSC.

Du *et al.*^120^ added an amino-acid or glycine self-assembled film to the SnO_2_-ETL at a reduced temperature, serving as a buffer layer to enhance the performance of the SnO_2_-based PSCs. In reality, the lattice mismatch between the SnO_2_ and perovskite layer was modulated by the buffer layer. Additionally, the interaction between SnO_2_ and the perovskite layer at the interface was improved by the electrostatic interactions between the amino group and perovskite framework. A schematic illustration of the SnO_2_-based PSC device architecture is shown in Fig. 7.

This resulted in the reduced recombination of charge carriers and improved charge carrier transport efficiency. They reported that the SnO_2_-based PSC modified with glycine had a maximum efficiency of 20.68%, FF of 0.78%, *V*_OC_ of 1.10 V, *J*_SC_ of 24.15 mA cm^−2^ and SnO_2_ or glycine may serve as an effective electron buffer layer for extremely efficient PSCs, as seen by the improved efficiency. Additionally, ternary metal oxides have superior characteristics compared to their binary counterparts. In ternary metal oxide materials, the ratios of the cations can be changed, and consequently their optoelectronic characteristics such as electric resistivity and bandgap can also be controlled. Therefore, ternary metal oxides, such as Zn_2_SnO_4_ (ZSO), SrTiO_3_ and BaSnO_3_,^121^ are potential ETL materials for developing highly efficient PSCs. Among the ternary metal oxides, ZSO has the best features, including strong electron mobility (10–30 cm^2^ V^−1^ s^−1^), broad optical bandgap (3.8 eV), and appropriate conduction band edge, making it a desirable electrode choice for PSCs.^121^ Oh *et al.* reported the fabrication of ZSO ETL-based PSCs with the PCE of 7% for the first time. Later, Shin *et al.* reported a novel technique for creating ZSO nanoparticles for solar applications. The produced ZSO nanoparticle-based PSCs exhibited a PCE of 15.3%.^122^ Subsequently, a solution-processed ZSO-film was employed by Jung *et al.*^123,124^ as an ETL in a PSC, resulting in a record-breaking efficiency of 20.02%. Recently, Zheng *et al.*^125^ created a Zn_2_SnO_4_ single crystal using a straightforward, affordable hydro-thermal synthesis process. The particles size and shape of the ZSO single crystal were controlled in the proposed method based on the duration of the hydrothermal reaction. Additionally, the ZSO-based perovskite solar cell displayed an elevated PCE of 18.32% together with a high *J*_SC_ of 24.79 mA cm^−2^. Moreover, the device remained stable even after 15 days in air with a humidity level of 20%. Thus, ZSO exhibits great potential as an ETL candidate for manufacturing exceptionally efficient photovoltaic devices, as demonstrated by all the above-mentioned findings.

Recently, ZSO was employed as an ETL in PSCs by Sadegh *et al.*^126^ They employed the chemical bath deposition (CBD) approach to modify the surface of the ZSO layer. The density and surface shape of the perovskite film were changed by CBD. Therefore, a perovskite layer with high surface exposure and enlarged grains was produced by CBD. The decrease in losses was caused by the recombination of charge carriers. These modifications significantly increased the charge extraction at the ETL/perovskite interface and successfully inhibited the trap-assisted recombination. Consequently, the photovoltaic performance was also improved simultaneously. The highest PCE of 21.3% was shown by the ZSO (ETL)-based PSCs treated with CBD. Specifically, the assembled device exhibited remarkable stability, maintaining 90% of its original PCE even after 1000 h of continuous illumination. New types of Sn-based materials are continuously being developed to enhance the performance of PSCs according to ongoing research.

**Fig. 8:**
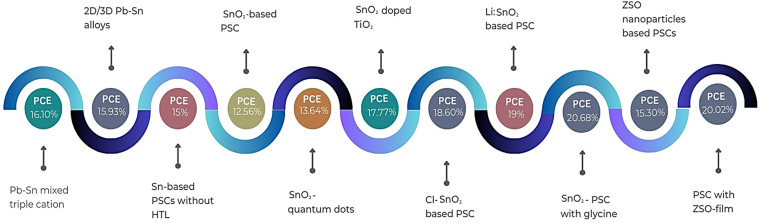
Progress in tin-based PSCs.

In conclusion, the exploration and development of Sn-based PSCs have emerged as a promising avenue in the field of photovoltaics, addressing the environmental concerns associated with their Pb-based counterparts. The intrinsic properties of Sn, coupled with its compatibility with various metal oxides and organic ligands, have paved the way for innovative device architectures and enhanced photovoltaic performances, as shown Fig. 8. The incorporation of diverse strategies, from the use of bulky organic ligands to the modulation of ETL materials, underscores the versatility and potential of Sn-based perovskites, as summarized in Table 4. Notably, the endeavors of researchers have showcased the potential of mixed cations, organic cations, and ETL modifications in achieving impressive PCEs. The recent advancements in ternary metal oxides, particularly ZSO, further highlight the continuous evolution of Sn-based PSCs towards achieving both high efficiency and stability. In this case, although challenges persist, especially regarding the oxidation of Sn and the synthesis of large-area films, the collective efforts of the scientific community signal a bright future for Sn-based PSCs.

**Table 4:** Progress in tin-based PSCs

Perovskite	Methodology	Significance/improvements	References
Pb–Sn mixed triple cation	Bulky organic ligands inclusion	Increase in spin orbit coupling (SOC) and out-of-plane photoinduced bulk polarization	105
2D/3D Pb–Sn alloys	Introduction of PEA^+^ (bulky organic cations) using solvent optimization	Growth-oriented orientations and SOC modulation resulting in photoinduced bulk-polarization	106
Sn-based PSCs without HTL	Use of natural antioxidant, *i.e.*, uric acid (UA)	Uric acid reduces oxidation and promotes carrier recombination	114
SnO_2_-based PSC	Strong pulsed light for quick annealing & inclusion of di-iodomethane alkyl-halide (CH_2_I_2_)	Enhanced surface characteristics of the perovskite layer	115
PSC with SnO_2_-quantum dots as ETL	Creation of SnO_2_ quantum dots using low-temperature solution processing	Better electron extraction and hole-blocking capabilities	116
SnO_2_-doped TiO_2_-based perovskite device	Tin-doped TiO_2_ ETL material preparation using hydriodic acid (HI)	Improved electron mobility and flaw suppression	117
PSC based on Cl-SnO_2_ and SnO_2_ as ETL	Implementation of compact and ultra-thin SnO_*x*_ coatings produced from SnCl_4_	Enhanced electron coupling, defect passivation, and energy level alignment	118
PSC based on Li:SnO_2_-ETL	Addition of LiCl to SnO_2_ ETL using a straightforward low thermal procedure	Increased conductivity of SnO_2_ enhancing charge transport and preventing charge recombination	119
SnO_2_-based PSC modified with glycine	Addition of an amino-acid or glycine self-assembled film onto the SnO_2_-ETL	Improved interaction between SnO_2_ and perovskite layer at the interface	120
ZSO nanoparticle-based PSCs	Creation of ZSO nanoparticles for solar application	Improved the stability of perovskite material	122
PSC with solution processed ZSO-film as ETL	Use of solution processed ZSO-film	Record-breaking efficiency	123
ZSO-based perovskite solar cell	Creation of a single crystal of Zn_2_SnO_4_ using hydro-thermal synthesis	Controlled particle size and shape of the ZSO single crystal	125
ZSO (ETL)-based PSCs treated with CBD	Chemical bath deposition (CBD) approach to modify the surface of the ZSO layer	Enhanced charge extraction at the ETL/perovskite interface and reduced trap-assisted recombination	126

## Mixed Sn–Pb PSCs

Pb-free perovskites have resolved the toxicity issue of lead-based perovskites, but their performance in practical applications is inferior to that of their Pb-based counterparts owing to their poor efficiency and stability. Thus, by combining lead and tin in perovskite structures, tin–lead perovskites may strike a balance between reduction in toxicity and obtaining excellent efficacy and stability.^127^ However, Sn-based perovskites solidify before Pb-based perovskites owing to the disparity in their crystallization rates. Accordingly, the Chen, Liu, and Guo research groups have made attempts to control the crystallization rates of these materials and promote vertical crystal growth. This approach aims to mitigate nonuniform growth, which results in the formation of numerous traps in the film that impede carrier transport.^128^ The device quality is increased by the improved crystallinity, which lowers the perovskite residual stress (or strain).^129^ An intriguing observation is that the bandgap of the material is narrowed to the range of 1.2–1.3 eV, which is smaller than that of perovskites based solely on lead or tin. This can be attributed to the “bowing effect” caused by the sharing of the octahedral cage between lead and tin in the perovskite structure.

The first Sn–Pb PSCs were reported in 2014 by the Hayase and Kanatzidis research groups, with efficiencies of 4.18% and 7.37%, respectively.^130,131^ To date, the Wakamiya group reported the greatest efficiency of 23.6%, surpassing the Hayase group's most recent achievement of 23.2%.^132^ Presently, attempted have been devoted to improving the effectiveness of Sn–Pb PSCs in a manner comparable to that of lead- and tin-based PSC investigations including n-doping of self-p-doped perovskite, surface creation of 2D perovskite, and additive for antioxidation.^133,134^ The special qualities and prospective uses of Sn–Pb PSCs are covered in the following section.

**Fig. 9:**
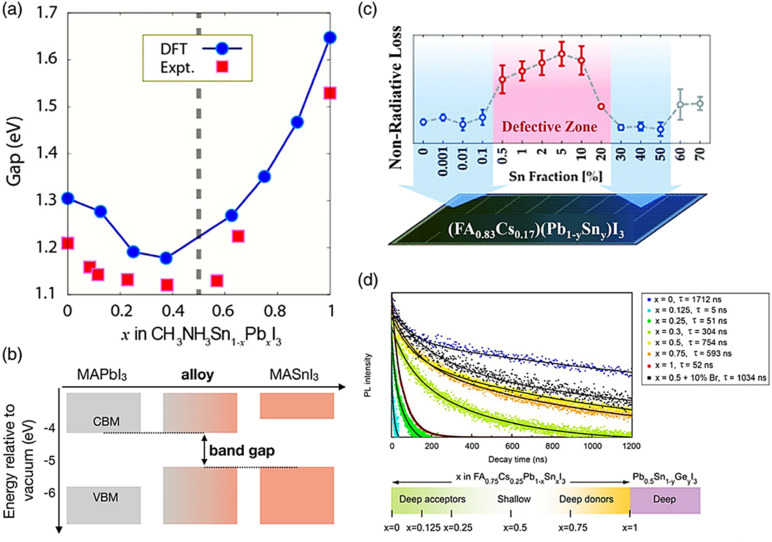
(a) Variation in the bandgap of Sn–Pb perovskite with changes in the Pb and Sn ratio in the B site. (b) Schematic illustrating how the bandgap is formed in Pb–Sn alloy perovskite. (c) Substituting Pb with Sn in perovskite leads to the formation of defect sites, causing nonradiative voltage loss. (d) Time-resolved photoluminescence spectra are obtained for FA_0.75_Cs_0.25_Sn_*x*_Pb_1−*x*_I_3_ perovskite layers with varying Sn amounts.^132^ (b) Ref. 133. (c) Ref. 134. (d) Ref. 135.

Fig. 9a depicts the bandgap bowing effect, demonstrating that a distinct alteration in the bandgap that occurs when Sn and Pb perovskites are alloyed. This effect leads to a reduction in the bandgap below that of each individual pure composition.^135^ The variation in both the energy level and lattice strain contribute to the bandgap bending of alloy perovskites. The band edge formations are mostly caused by inconsistencies in the energy levels of the atomic orbitals of Sn and Pb. The VBM and CBM of Sn perovskite are shifted upwards (Fig. 9b), as described in the section on Sn-based perovskites.^136^ These discrepancies cause Sn–Pb perovskites to have a narrow bandgap. Smaller Sn has an indirect influence on the bandgap value by causing the octahedron to tilt and the lattice to compress.^137^ Changes in the A- and/or X-site ions have an impact on the bandgap values in lead- and tin-based perovskites, as covered in the prior sections. The bandgaps of mixed Sn–Pb perovskites exhibit variations influenced by the organic cation and halide present. The lowest bandgap and ABX_3_ perovskites was reported when the ratio of Sn to the B element (Sn/Pb) was in the range of 40% to 70%.^138^

Also, the bandgap can be controlled, the defect can be reduced, and the oxidation can be suppressed. As shown in Fig. 9c, the Snaith group showed that the Sn concentration in perovskite in the range of 0.5% to 20% of the metal content caused faults, but an Sn content in the range of 30% to 50% recovered the optoelectronic quality.^139^ Trap sites were created and the photoconductivity, photoluminescence lifespan, and photoluminescence quantum efficiency all decreased with the inclusion of a modest concentration of Sn. The Sargent group demonstrated that deep-level traps were formed when the Sn concentration was less than 30%.^140^ Alternatively, the 50% Sn mixed alloy showed extended carrier lifetimes and improved defect tolerance without deep traps (Fig. 9d). The oxidation level varied depending on the Sn concentration. Sn^4+^ is quickly produced on the perovskite surface, according to the theory of the Angelis group that Sn-poor conditions enhance the oxidation of Sn because it functions as a dopant.^141^

Sn is more readily oxidized than Pb, which leads to more flaws and severe oxidation. However, it has been shown that increasing the stability and film quality of perovskites requires an Sn concentration of roughly 50%. The advantage of having a low bandgap opens up a range of applications, including photodetectors and tandem solar cells,^142^ given that the bandgap of Sn–Pb perovskites with 50% Sn content is close to 1.2 eV and may potentially attain sufficiently high efficiency (32.74%). Recently, the wide absorption spectrum (1000 nm) of Sn–Pb mixed perovskites has been applied in several investigations on photodetectors. These photo-detectors struggle to detect light in the NIR region because the absorption spectrum of Pb-based perovskite is restricted to the range of 300 to 800 nm.^143^ Thus, to extend its absorption spectrum to 1000 nm, organic bulk-heterojunction (BHJ) layers capable of absorbing in the near-infrared (NIR) region are deposited on the perovskite film.^144^ In organic photovoltaics, the morphology of the BHJ layer is a critical in determining the device performance, particularly impacting its light absorption capabilities. This layer, typically a blend of electron-donating and electron-accepting organic materials, creates a nanoscale network of interpenetrating phases, each with distinct roles in the device operation. The surface morphology of the BHJ layer is pivotal for several reasons. Firstly, it defines the interfacial area between the donor and acceptor materials, which is crucial for effective exciton dissociation. A larger interfacial area means more opportunities for light absorption and exciton generation. This is particularly important in the context of extending the absorption spectrum, given that a greater interfacial area results in more effective light harvesting, including in the NIR region. Additionally, the morphology dictates the pathways available for charge transport. An optimal network with efficient pathways ensures that the photogenerated carriers (electrons and holes) can reach their respective electrodes effectively, contributing to the overall current of the device. The morphology also affects the exciton diffusion length, which is the distance which excitons have to travel to recombine.^145,146^ A favorable morphology with appropriate domain sizes increases the likelihood of excitons reaching a donor–acceptor interface for dissociation, thereby enhancing the efficiency of light absorption and conversion.

Another crucial aspect influenced by morphology is the rate of recombination. Poorly connected phases can lead to increased charge recombination, where electrons and holes recombine prematurely, leading to energy loss. Thus, optimizing the morphology is key to minimizing these recombination losses. Furthermore, the way light interacts with the active layer is significantly influenced by the surface morphology of the BHJ layer. This morphological structure can scatter or trap light, directly affecting the absorption profile of the material.^147^ For instance, aggregated domains or irregular structures in the BHJ layer may enhance light trapping, which is particularly beneficial for extending the absorption into the NIR region. This effect is critical for devices designed to absorb a broader spectrum of solar radiation, including wavelengths that standard photovoltaic materials may not efficiently capture. Moreover, the morphological features at the interfaces with other layers, such as electrodes, are vital in impacting charge injection and extraction processes. An optimal morphology ensures efficient charge transport and extraction, minimizing the energy losses at these crucial interfaces. Therefore, achieving the appropriate morphology is essential for efficient device operation, particularly in ensuring that the perovskite layer and the BHJ layer work synergistically.

**Fig. 10:**
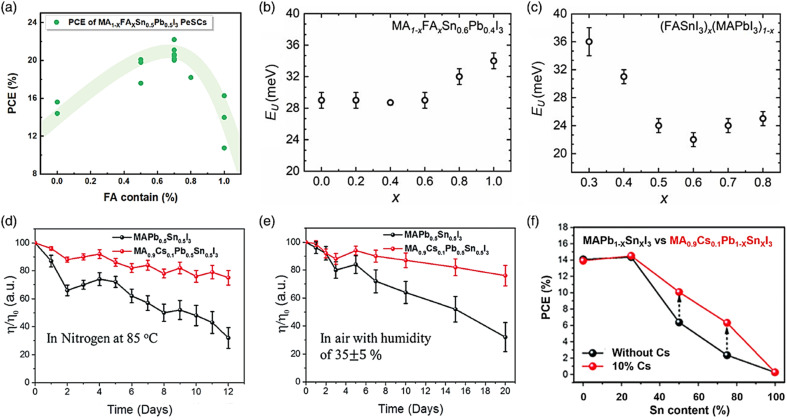
(a) PCE of MA_1−*x*_FA_*x*_Sn_0.5_Pb_0.5_I_3_ PSCs. UE in relation to the changing perovskite composition for (b) MA_1−*x*_FA_*x*_Pb_0.4_Sn_0.6_I_3_ and (c) (FASnI_3_)_*x*_(MAPbI_3_)_1−*x*_. (d–f) Thermal and air stability test conducted on the device at 85 °C with 10% Cs addition. Stability and efficiency increase with the inclusion of Cs in Sn_*x*_Pb_1−*x*_ PSCs. (b and c) Ref. 143. (d–f) Ref. 144.

However, the NIR detectivity of Pb-based perovskite photodetectors in combination with BHJ is poor. Fortunately, better detectivity and sensitivity can be achieved by Sn–Pb-based perovskite, which absorbs NIR light.^148^ Perovskite tandem solar cells are also used. A rear broad-bandgap solar cell that absorbs high-energy photons and a front smaller-bandgap solar cell that absorbs low-energy photons make up a perovskite tandem solar cell in most cases. To date, the top cells are generally made of organic, CIGS, and Si solar cells,^149^ which are further explained in the next section. Sn–Pb-based perovskite is a possible contender for the fabrication of the top cells^150^ due to its high efficiency, low production cost, and solution processability. Thus, to achieve a remarkable efficiency of over 20% in Sn–Pb perovskite solar cells (PSCs), the composition of the perovskite material, specifically the mixed A cation, needs to be fine-tuned, as demonstrated in Fig. 10a. The major materials utilized are FA and MA, with a small quantity of Cs added on occasion. The best efficiency was observed when the FA-based perovskite was comprised of 30 mol% of MA, as demonstrated in the efficiency trend of the ASn_0.5_Pb_0.5_I_3_ PSCs. The Podraza group discovered a connection between the Urbach energy (UE) and the increased efficiency of mixed cation Sn–Pb PSCs. By using photothermal deflection spectroscopy, they evaluated the UE of perovskites with diverse cation compositions. The low UE and *V*_OC_ of thin-film perovskites exhibit a clear correlation, with the reduction in UE happening in conjunction with a positive decline in the *V*_OC_ deficit. They demonstrated that a lower UE and *V*_OC_ reduction occurs when FA and MA are blended in an equivalent ratio (Fig. 10b and c, respectively).^151^

A well-known substance, inorganic Cs, considerably increases the moisture and light stability of perovskite films.^152^ The Jen group established that Sn–Pb-based perovskites with partially substituted MA^+^ or FA^+^ for Cs^+^ exhibit a slowed crystallization rate to promote the creation of homogeneous films.^153^ In particular, in compositions including high concentrations of Sn, the device stability and performance were improved (Fig. 10d–f). The Sn–Pb perovskite has an intriguing property, where its bandgap changes randomly when the mixed A-site is present. Materials having a smaller A site predominate, APbI_3_ develops a bigger bandgap, and ASnI_3_ exhibits the reverse trend.^154^ However, there is no obvious regularity in the case of Sn–Pb perovskite due to variations in the extent of orbital binding and deformation of the perovskite lattice caused by the mixed B-site.^155^

**Fig. 11:**
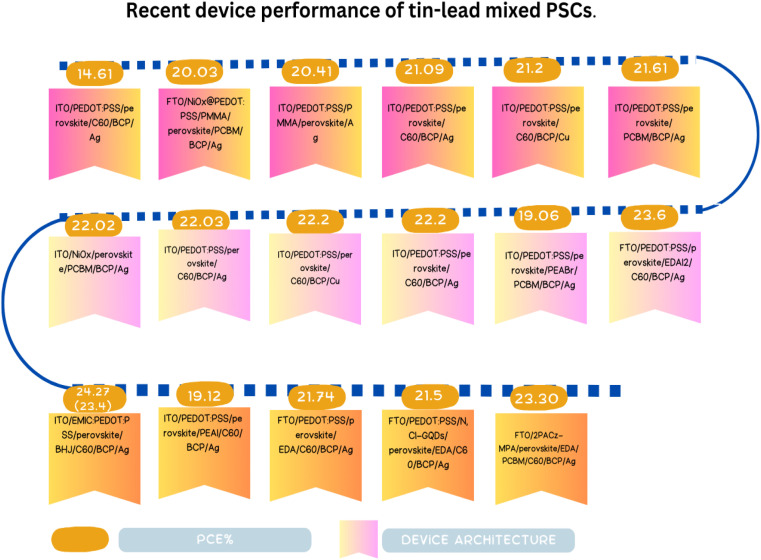
Recent device performance of tin–lead mixed PSCs.

In summary, the evolution of mixed Sn–Pb PSCs underscores the relentless pursuit by the scientific community to merge environmental sustainability with optimal device performance. The amalgamation of tin and lead in perovskite structures has unveiled a promising pathway, bridging the gap between the high efficiency of Pb-based perovskites and the environmental benignity of their Sn-based counterparts, as shown in Fig. 11. The intricate interplay among the “bowing effect,” crystallization rates and defect dynamics has been pivotal in shaping the optoelectronic properties of these mixed perovskites. Noteworthy strides by various research groups have illuminated the potential of manipulating the Sn concentration to enhance the film quality, suppress oxidation, and fine-tune the bandgaps, opening doors to diverse applications such as photodetectors and tandem solar cells. The extended absorption spectrum of Sn–Pb perovskites, especially in the NIR region, further accentuates their versatility and potential in photodetection, as summarized in Table 5. As this field continues to flourish, the insights gleaned from these studies will undoubtedly serve as foundational pillars, guiding future endeavors in the field of perovskite solar cells.

**Table 5:** Progress in Pb–Sn based PSCs

Perovskite type	Methodology	Significance/improvements	References
Tin–lead (Sn–Pb) perovskites	Combining lead and tin in perovskite structures to balance toxicity reduction with efficacy and stability	Reduction in toxicity with maintained efficacy and stability; Sn-based perovskites solidify before Pb-based due to their difference in crystallization rate	127
Tin–lead (Sn–Pb) perovskites	Control of crystallization rates to promote vertical crystal growth and mitigate nonuniform growth	Improved device quality due to enhanced crystallinity, reducing residual stress in the perovskite	128
Tin–lead (Sn–Pb) perovskites	Studying the bandgap bowing effect in Sn and Pb alloyed perovskites	Narrower bandgap due to ‘bowing effect’, leading to variations influenced by the organic cation and halide present	135
Tin–lead (Sn–Pb) perovskites	Adjusting Sn concentration in perovskites to control defect formation and oxidation	Sn concentration between 30% and 50% recovers optoelectronic quality; lower concentrations create deep-level traps	139
Tin–lead (Sn–Pb) perovskites	Fine-tuning the composition of the perovskite material, specifically the mixed A cation	Higher efficiency achieved with specific ratios of FA and MA; correlation between Urbach energy and efficiency	150 and 151
Tin–lead (Sn–Pb) perovskites	Partially substituting MA^+^ or FA^+^ with inorganic Cs in Sn–Pb-based perovskites to enhance film stability	Increased moisture and light stability; homogeneous film formation, especially in high Sn concentrations	153

## Germanium-based PSCs

Germanium (Ge), another member of group IV, can also serve as a substitute for Pb or Sn in the creation of perovskites. However, Ge-based perovskites exhibit distinct characteristics compared to their Pb^2+^ and Sn^2+^ counterparts, primarily because Ge is a significantly lighter element. Due to the propensity of Ge^2+^ to oxidize to the more stable Ge^4+^, germanium-based perovskites tend to be less stable than tin-based ones. Furthermore, its considerably smaller radius (73 pm) does not align well with the ABX_3_ structure of perovskites based on Goldschmidt's tolerance factor. These perovskites display broader optical bandgaps exceeding 1.6 eV and markedly different energy levels. For instance, the conduction band minimum (CBM) of FAGeI_3_ is 3.15 eV, whereas that of FAPbI_3_ and FASnI_3_ is 4.0 eV and 3.79 eV, respectively.^156,157^

**Fig. 12:**
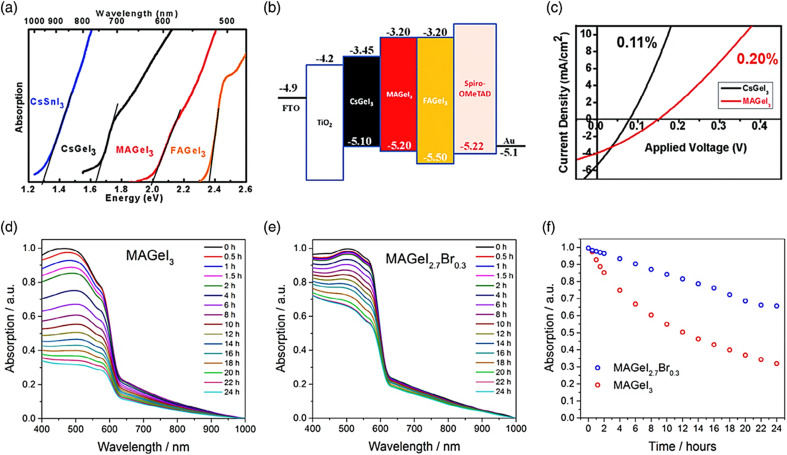
(a) Absorption characteristics of MAGeI_3_, CsGeI_3_ and FAGeI_3_ in comparison with CsSnI_3_. (b) Schematic energy level diagram of CsGeI_3_, FAGeI_3_ and MAGeI_3_. (c) *J*–*V* curves of photovoltaic devices fabricated with different Ge halide perovskites. Time-resolved UV-vis measurements to investigate the ambient stability of the germanium perovskite samples of (d) MAGeI_3_ and (e) MAGeI_2.7_Br_0.3_ and (f) absorption intensity at 510 nm *versus* time. (a–c) Ref. 149. (d–f) Ref. 151.

Despite the numerous theoretical investigations on Ge-based perovskites, the use of germanium-based perovskites in solar cells has been scarcely documented.^158^ The first germanium-based PSCs with CsGeI_3_, MAGeI_3_, or FAGeI_3_ photoactive layers were reported in 2015.^157^Fig. 12 illustrates that all the produced PSCs, including CsGeI_3_ solar cells, performed poorly, achieving a PCE of less than 0.20%. This was attributed to the fact that the bandgaps of MAGeI_3_ and FAGeI_3_ are too wide to effectively absorb light. In 2018, by substituting 10% of the iodide in germanium-based PSCs with bromide, the PCE increased to 0.57%.^159^ These subpar performances appear to originate from the formation of imperfect crystals, instability, and poor surface configuration of Ge-based perovskites.^157^

Mixed tin and lead-based perovskites (Sn–Pb) are considered alternatives to lead perovskites. However, given that Ge is non-toxic and theoretically offers a performance comparable to lead PSCs, it should also be considered. The simulation results by Raman *et al.* suggest that Ge–Pb-based PSCs using an MA(Pb,Ge)I_3_ light absorber can potentially achieve efficiencies of up to 30%.^160^ Nevertheless, there have been limited studies on Ge–Pb devices due to the instability of the Ge–Pb perovskite crystal (tolerance factor of >1). Another simulation-based study indicated that ternary B-site mixed cations, including Pb, Sn, and Ge, can be used to develop stable and efficient devices.^161,162^ Experimental studies have identified In, Cu, and Zn as feasible substitutes for reduced-lead perovskites.^163^ Currently, germanium-based perovskites are primarily used in combination with tin-based perovskites due to performance concerns.^164^

## Polymer-based PSCs

Researchers have been focused on improving the durability of PSCs by interfacial modification, device encapsulation and inverted orientation, together with other methods.^165^ According to reports, intrinsic alteration may also increase the stability of perovskites, particularly by reducing moisture–corrosion.^166^ The active layer, such as the ETL, HTL, photovoltaic operational layer and buffering intermediate layer, is often the location of the interfacial alteration.^167^ However, the perovskite/perovskite contact, which is called the intergranular interface, has a higher defect density, which makes it easier for moisture to penetrate the perovskite layer.^168,169^ Thus, to optimize the morphology, charge transfer, and separation in the perovskite layer, the crystallinity at the intergranular interface must be increased.^170^ An excellent photovoltaic performance is achieved as a result of better charge separation and transportation in the perovskite film. Consequently, PSCs with perovskite intergranular interfaces that are resistant to moisture display enhanced stability.^171^

Researchers are focused on improving the interactions between the perovskite layer grains, such as acid-base, stacking, electrostatic interactions, and hydrogen bonding, to provide moisture-resistant intergranular interface.^168^ The moisture-resistant intragranular interface in perovskite solar cells significantly impacts the exciton depletion and electron/hole mobilities, which are essential for the overall efficiency of the cells. Firstly, regarding exciton depletion, grain boundaries in the perovskite materials are crucial. These boundaries, when stable and moisture-resistant, introduce fewer deep defect states in the band gaps, maintaining high electronic quality. Grain boundaries can provide additional pathways for exciton dissociation and charge separation, enhancing the exciton depletion.^145^ Furthermore, the ordered perovskite/perovskite heterojunctions, formed through molecular modification of the perovskite layer, aid in efficient exciton dissociation and charge carrier transport in the grains. The moisture resistance at these interfaces is critical for maintaining the integrity of the perovskite layer and preventing the entry of water, which reduces the charge recombination, and thereby effectively depletes excitons. Regarding the aspect of electron and hole mobilities, the ETL interface plays a significant role. Moisture-resistant modifications at the perovskite/ETL interface, such as incorporating bifunctional molecules and fullerene derivatives, optimize the electronic structure and passivate recombination processes. This results in enhanced electron mobility by reducing the number of trap sites and improving the interface band alignments. Similarly, at the perovskite/HTL interface, functional modifications can facilitate improved hole extraction and electron blocking, leading to better hole mobility in the perovskite layer. The development of ordered perovskite/perovskite heterojunctions also influences the charge carrier mobilities, where the well-aligned interlayers and minimized defects in these heterojunctions promote efficient electron and hole transport.

Substituting phenethylamine (PhCH_2_CH_2_NH_2_) with methylamine (CH_3_NH_2_) may improve the hydrophobic interactions and stacking. Additionally, formamidine is used to replace methylamine (CH_3_NH_2_) to increase the hydrogen bonding contact. Consequently, the stability and PCE of PSCs are improved. Many polymers, including polyethylene oxide (PEO), polymethylmethacrylate (PMMA), polyethyleneimine (PEI) and polyvinylpyrrolidone (PVP) display significant intergranular interactions as a result of the presence of many active sites.^172,173^ Also, polymer-based PSCs display great stability and enhanced PCE because of the presence of significant intergranular contacts.^174^ To date, in all the reported polymers, their curly macromolecular arrangement has been altered to modify the intergranular contacts of the perovskite. This is because their arrangement will have a negative effect, such as reducing the perovskite crystallinity or diminishing its photoelectric property. Researchers are actively developing improved polymers, specifically dendritic polymers or dendrimers, to address existing challenges. These 3D spherical polymers have generated significant interest due to their ability to make slight configuration adjustments when interacting with the perovskite grain surfaces, thereby preventing local aggregation in their linear macromolecular structure.^175^ Consequently, the crystallinity of dendrimers is enhanced. Therefore, the stability and effectiveness of perovskite devices are significantly enhanced by the use of dendritic polymers.

Consistent with this, Du *et al.* created a novel molecular roadmap to illuminate the efficacy of PSCs. The intergranular perovskite contact was successfully regulated by the suggested model, which enhanced the PCE. Polyamidoamine (PAMAM) dendrimers were employed by Du *et al.* as a template for the dendritic crystallization process that formed the perovskite.^176^ PAMAM contains methyl esters at its molecular perimeter, and these groups have tremendous potential to interact with the grain surfaces of the perovskites. These interactions, likely involving chemical bonding or other molecular associations, are poised to significantly influence the surface morphology of the perovskite layer. The primary objective of this interaction between the PAMAM functional groups and the perovskite grains is to enhance the intergranular interface interactions, strengthening the overall structure of the perovskite layer. These interactions with PAMAM induce alterations in the chemical structure of the active perovskite layer, which affect the organization of the perovskite grains, leading to changes in the surface morphology and the interfacial interactions between the grains. The aim is to enhance the compactness and uniformity of the perovskite film, which includes reducing the number of defects such as pinholes and bolstering the intergranular connections.^146^ These chemical and morphological modifications in the perovskite layer can substantially impact the performance of the device. A more compact and uniformly distributed perovskite film, achieved through the PAMAM interactions, facilitate improved charge separation and reduced recombination rates, thereby enhancing the overall stability and efficiency of the device. The fortification of the intergranular interactions is especially vital for the durability and performance of perovskite solar cells. Furthermore, the interaction between the methyl esters in the PAMAM dendrimers and perovskite grain surfaces results in notable changes in the chemical structure of the active layer. This alteration positively influences the surface morphology, leading to enhancements in device performance. The methyl esters in PAMAM interact with the perovskite grain surfaces, forming bonds through its amino and carbonyl groups. The PAMAM dendrimers act as a dendritic crystallization framework, guiding the formation of the perovskite layer. This process crosslinks the perovskite grains, substantially strengthening the intergranular interfacial interactions.

**Fig. 13:**
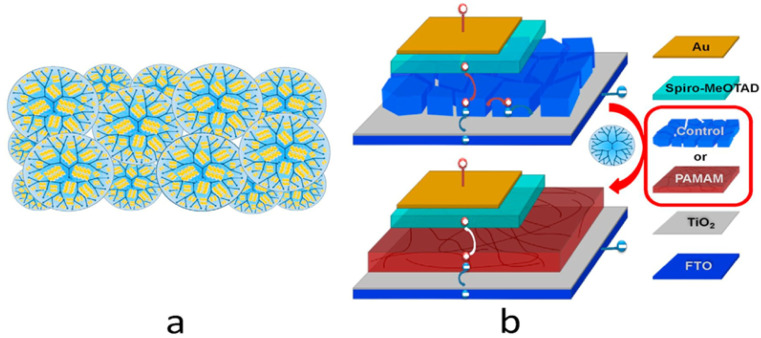
(a) Schematic depiction of perovskite morphology regulation by PAMAM dendrimers. (b) Device structure of unmodified and PAMAM-modified perovskite photovoltaic devices.^176^

The result is a marked improvement in the perovskite phase morphology, which is characterized by a reduction in the number of grain boundaries and elimination of pinholes. These morphological improvements caused by PAMAM modification synergistically enhance the power conversion efficiency and stability of perovskite solar cells. The increase in the short-circuit current density, leading to a significant enhancement in power conversion efficiency, is chiefly attributed to the improved perovskite morphology, and in particular, the robust intergranular interactions facilitated by PAMAM. Because of this, the dendritic-polymer backbone of the perovskite grains exhibits high intergranular interfacial contacts. Additionally, the phase shape of the perovskite is significantly improved by getting rid of the pinholes and reducing the number of grain boundaries. To create a homogeneous surface, the compact perovskite layer and non-pinhole dendritic PAMAM crosslinked the perovskite grain, strengthening the contacts at the inter-granular interface. This resulted in a substantial PCE of 42.6% for the unencapsulated PSCs using the PAMAM polymer (dendritic) backbone under ambient conditions. The PAMAM-modified device also retained 73% of its original PCE after 400 h. The major factor in achieving a high PCE is the enhancement in the perovskite intergranular interactions by PAMAM modification. A schematic depiction of the PAMAM dendrimers controlling the perovskite morphology is shown in Fig. 13, together with the device architecture of a PAMAM-modified PSC.

Interlayers also significantly contribute to increasing the PCE of PSCs. Recently, poly-electrolytes have been shown to have an impact on the device performance when utilized as buffer layers in both n-type substrate (N–I–P) and p-type substrate (P–I–N) geometries, according to Kang *et al.*^177^ To create the buffer layers, they employed non-conjugated polymer electrolytes (NPEs) with a PEI backbone and a variety of counterions, including tetrakis(imidazole) borate (BIm_4_^−^). bromide (Br^−^), and iodide (I^−^). Additionally, the size of the counterion affects the performance of perovskite solar cells. The non-conjugated polymers produced electric dipoles at the NPE/metal electrode interface, which could be used to adjust the energy levels and work functions of the electrodes. Consequently, in the N–I–P and P–I–N configurations, the solar cell incorporating the NPE buffering layer displayed a PCE of 14.71% and 13.79%, respectively.

At the interface between the perovskite and electrode, the HTL layer generally removes the holes and inhibits the recombination of charge carriers, which affects the PCE of PSCs. Therefore, developing HTLs is equally crucial for creating high-performance PSCs.^178^ In this case, due to its good film shape, high conductivity, and ability to be processed in a solution at low temperatures, polyethylenedioxythiophene (PEDOT):polystyrenesulfonate (PSS) has been one of the most often utilized HTL in inverted PSCs thus far.^179^ However, despite its benefits, the main limitations of the PEDOT:PSS-based HTL in PSCs is the acidic nature of PEDOT:PSS.^180^ Thus, numerous researchers are looking for novel strategies to advance the PV device performance by addressing the drawbacks of PEDOT:PSS HTLs.^181^

**Fig. 14:**
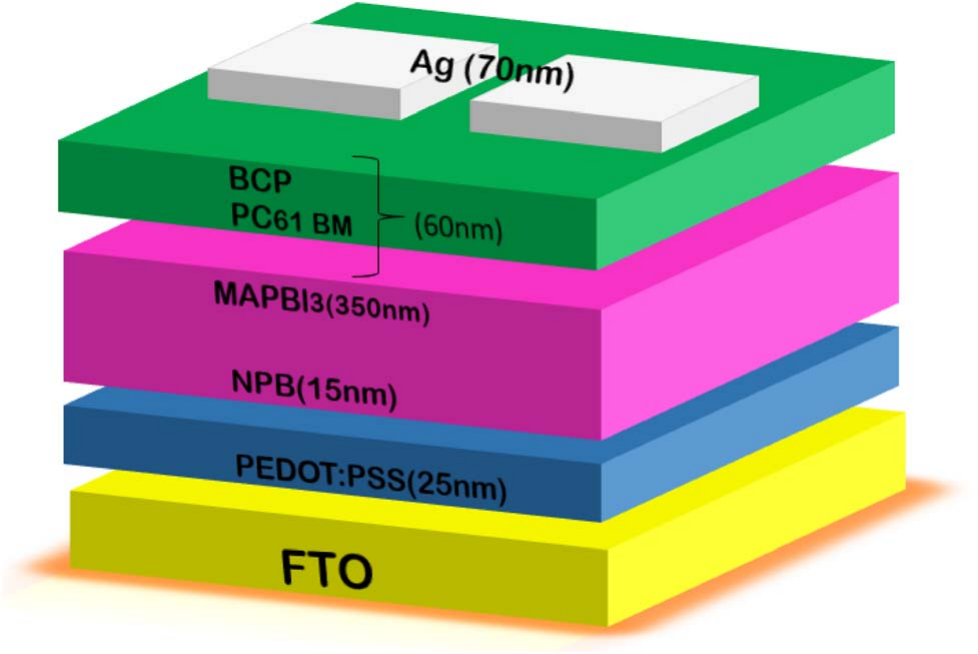
Structure of the PSC based on NPB.

Recently, a unique method for synthesizing new HTLs using the readily available copper thiocyanate (CuSCN) was suggested by Xu *et al.*^182^ According to their findings, adding CuSCN to PEDOT:PSS, and then annealing it at a low temperature lowers the energy barrier and improves the charge extraction yield, while also having an acidic nature. Consequently, the PCE of the CuSCN-modified PEDOT:PSS HTL-based PSC was 15.3% at 1.0 V, which was 16% higher than that of the PEDOT:PSS HTL-based PSCs. Additionally, the decreased acidity produced exceptional longevity, as shown by the retention of 71% of the original PCE of the device after 175 h of exposure to N_2_ under full sun. *N*,*N*′-Bis-(1-naphthaleny)-*N*,*N*′-bis-phenyl-(1,1′-biphenyl)-4,4′-diamine (NPB) is a tiny triphenylamine-based chemical, which Ma *et al.*^183^ added to a perovskite solar cell as a multifunctional buffer layer to further improve its PCE. The device configuration of the NPB-based PSC is shown in Fig. 14.

According to their findings, the use of NPB as a buffer layer lowered the amount of pinholes and imperfections in the perovskite films and modified the energy imbalance between the perovskite structure and PEDOT:PSS film. Due to the diminished defects and pinholes at the interface of the perovskite/PEDOT:PSS layers, the electron–hole recombination was severely constrained in the NPB-modified device. Consequently, the PCE of 18.4% was shown by the NPB-modified PSC. The same device displayed a PCE of 14.4% under UV light without hysteresis and great stability. According to the suggested method, creating the advanced generation of effective, exceptionally stable and flexible PSCs may rely heavily on NPB as a buffer layer. This is because the NPB buffer layer plays a pivotal role in enhancing the surface morphology of the perovskite films. This improvement is evident in the scanning electron microscopy (SEM) images, which demonstrated a denser and more uniform film structure upon the introduction of NPB. The increased coverage and uniformity provided by the NPB layer are crucial in diminishing the presence of pinholes in the film. Secondly, the use of NPB affects the wettability of the perovskite layer. Contact angle measurements revealed a modest decrease in wettability with the incorporation of NPB. This reduction in wettability is associated with improved film quality, given that it aids in minimizing the number of pinholes and surface imperfections. The enhanced film formation due to the altered wettability contributed to a smoother and more consistent perovskite layer. Furthermore, the chemical interaction between NPB and the perovskite film was instrumental in suppressing the formation of defects, particularly at the interface with PEDOT:PSS.^184^ This chemical synergy not only enhanced the quality of the perovskite film but also contributed to a more integrated and coherent layer structure. Lastly, the presence of NPB helps reduce the interfacial defects that typically arise due to the chemical reaction between PEDOT:PSS and the perovskite precursor. In the absence of an NPB buffer layer, these reactions can lead to the formation of detrimental interfacial defects. However, the introduction of NPB effectively suppresses these defects, leading to an overall improvement in the film quality.

Thus far, nearly all the reported flexible PSCs have tiny surface areas. However, as the films inevitably experience reduced uniformity, it is widely recognized that the PCE decreases when the device area is scaled up to a large extent. Therefore, the performance of large-area flexible PSCs is directly influenced by the large-scale thin-film deposition process. Accordingly, wide-area manufacturing methods must be developed to create flexible PSCs with all their layers. In order to further reduce the cost of manufacturing, alternative technologies should be offered, which should ideally encourage the development of useful applications.

Table 6 summarizes the recent developments in polymer-based PSC. The exploration in polymer-based PSCs has unveiled a plethora of opportunities and challenges in the field of perovskite solar technology. The intrinsic modifications, especially at the intergranular interface, have emerged as a pivotal strategy to enhance both the efficiency and stability of PSCs. The innovative use of dendritic polymers, particularly PAMAM, has showcased the potential of molecular engineering in optimizing the morphology and performance of perovskites. Furthermore, the development of novel HTLs and buffer layers, such as CuSCN-modified PEDOT:PSS and NPB, respectively, have set new benchmarks in device efficiency and stability. However, as we venture into the domain of large-area flexible PSCs, the challenges of maintaining uniformity and efficiency at a larger scale become evident. Therefore, the future of polymer-based PSCs hinges on the development of scalable deposition processes and innovative materials that can maintain high performances across larger device areas.

**Table 6:** Recent developments in polymer-based PSCs

Category	Methodology/technique	Significance/improvements	References
Interfacial modification	Device encapsulation, inverted orientation	Enhanced durability	157, 176 and 177
Intrinsic alteration	Reducing moisture–corrosion	Increased stability of perovskites	158 and 178
Intergranular interface optimization	Enhancing crystallinity	Better charge separation and transportation; however, higher defect density	160–163, 179 and 180
Polymer-based PSCs	Use of PEO, PMMA, PEI, PVP	Enhanced resilience and PCE; however, may reduce the crystallinity or photoelectric property of the perovskite	164–166 and 181–185
Dendritic polymers	Use of dendritic polymers	Enhanced stability and effectiveness	167 and 186
PAMAM dendrimers	Polyamidoamine (PAMAM) dendrimers	Improved PCE to 42.60%, enhanced perovskite intergranular interactions	168
NPE buffer layers	Nonconjugated polymer electrolytes (NPEs)	Improved PCE in both N–I–P (14.71%) and P–I–N (13.79%) configurations	169
PEDOT:PSS HTLs	CuSCN-modified PEDOT:PSS HTL	Improved PCE to 15.30%, exceptional longevity. However PEDOT:PSS is acidic in nature	174
NPB buffer layer	NPB as a buffer layer	Reduced electron–hole recombination, improved PCE to 18.40%	175

## Halide double perovskite solar cells (HDPs)

Halide double perovskite solar cells (HDPs) are created by substituting one monovalent cation B^+^ and one trivalent cation B^3+^ for two Pb^2+^, with the formula of A_2_B^+^B^3+^X_6_. The general chemical formula for Pb halide perovskites can be described as APbX_3_, wherein A represents either an organic or inorganic cation (such as MA^+^, Cs^+^, and FA^+^), B signifies Pb^2+^, and X denotes a halogen (Cl^−^, Br^−^, and I^−^). The A-site cations occupy the cavities formed by eight [PbX_6_]^4−^ octahedra. With alternating [B^+^X_6_]^5−^ and [B^3+^X_6_]^3−^ octahedra, the double perovskites have a similar three-dimensional structure to Pb perovskites, and the A-site cations are located in the cavities created by the octahedra.

It is important to note that HDPs are much more stable and typically non or less hazardous than Pb-based perovskites (with the exception of Tl-based compounds). Two distinct B-site cations provide access to a broad variety of potential combinations and rich substitutional chemistry, which is equally significant. The choices for both the A cation and X anion are limited, with the A cation consisting mostly of Cs^+^ and CH_3_NH^3+^ (MA^+^) and the X anion consisting primarily of Cl^−^, Br^−^, and I^−^. Alternatively, the selection of the B-site cations is more flexible and may include Ag^+^, Na^+^, Li^+^, Au^+^, Bi^3+^, SB^3+^, In^3+^, Fe^3+^, and Tl^3+^. Given that the related HDPs are more desirable for phosphors or light-emitting diodes, other B^3+^ cations such rare-earth ions are not noted here.^185^ These components may be easily permuted and combined to produce hundreds of HDPs. Meanwhile, two distinct B-site metal ions give several potential for alloying and doping in HDPs by a variety of elements, thereby extending the broad family of HDPs and providing enormous prospects for HDP-based photovoltaics.

The study on Cs_2_AgBiBr_6_ in 2016 prompted significant interest in HDPs. Bein and coworkers created the first double perovskite solar cell devices in 2017 after resolving the poor solubility of the precursors in DMSO at 75 °C and spin coating thin films.^186^ They underlined that obtaining a pure Cs_2_AgBiBr_6_ double perovskite phase requires the use of a high annealing temperature (250 °C). Additionally, they created the first Cs_2_AgBiBr_6_ solar cell device with the conventional mesoporous structure and attained a PCE of 2.43%.^186^ These Cs_2_AgBiBr_6_-based devices impressively demonstrated exceptional stability under continuous illumination for 100 min or ambient settings for at least 25 days. Using Cs_2_AgBiBr_6_ single crystals as the precursor solution, we simultaneously produced a highly uniform and high-quality Cs_2_AgBiBr_6_ thin film made of single-layer nanocrystals. With the structure ITO/TiO_2_/Cs_2_AgBiBr_6_ (205 nm)/spiro-OMeTAD/Au, we further demonstrated the first planar Cs_2_AgBiBr_6_ solar cells. With a *V*_OC_ of 1.06, *J*_SC_ of 1.55 mA cm^−2^ and FF of 74%, the champion device exhibited a PCE of 1.22%.^187^ This planar device structure showed little hysteresis.

Thus far, a variety of deposition techniques has been used to explore the mechanisms that affect the PV performance of Cs_2_AgBiBr_6_ solar cells. Controlling the crystallinity, shape, orientation, thickness, phase purity, *etc.* of the film, which may affect the PCE of solar cell devices, is the driving force for the use of various manufacturing procedures.^188^ Low-pressure-aided solution processing was used by Xiao and colleagues to create planar solar cells with an optimum PCE of 1.44%.^189^ Cs_2_AgBiBr_6_ thin films were successfully created by Liu and colleagues using a sequential vapor deposition method. The device displayed a PCE of 1.37%, *V*_OC_ of 1.12 V, *J*_SC_ of 1.79 mA cm^−2^, and FF of 68%.^190^ They also emphasized the need for extra BiBr_3_ to produce stoichiometric Cs_2_AgBiBr_6_ double perovskite thin films. Yang and coworkers later verified this. The vapor-processed film displayed a deviating composition stoichiometry as a result of a greater loss of Br.^191^ After 350 h of ambient storage without encapsulation, the vapor-processed devices retained over 90% of their original PCEs. It was also utilized to create Cs_2_AgBiBr_6_ thin films with ultrasmooth morphology, micro-sized grains, and high crystallinity. Antisolvent dropping is a common technique for the fabrication of Pb-based perovskite solar cells, resulting in *V*_OC_ = 1.01 V, *J*_SC_ = 3.19 mA cm^−2^, and FF = 69.2%, and the resultant solar cells exhibited the optimum PCE of 2.23%.^192^

Additionally, these devices retained 90% of their original PCEs after 10 days of storage and hardly exhibited any performance loss when annealed for 60 min at 100 °C. Employing a dye-sensitized ETL or HTL is an intriguing method to boost the *J*_SC_ of Cs_2_AgBiBr_6_ solar cells. For instance, a device based on C-Chl-sensitized mesoporous TiO_2_ pushed the PCE to 3.11% by increasing the *J*_SC_ from 3.22 to 4.09 mA cm^−2^.^193^ Similarly, by sensitizing the TiO_2_ ETL with D149 indoline and adding Ti_3_C_2_T_*x*_ MXene nanosheets to Cs_2_AgBiBr_6_, the *J*_SC_ of the final device reached up to 8.85 mA cm^−2^. According to the external quantum efficiency (EQE) spectra, the dye increased the absorption of sunlight between 500 and 650 nm, which was the primary source of the enhanced photocurrent. Consequently, a very high PCE of 4.47% was attained.^194^ Additionally, after 1000 h of storage in air (approximately 20% relative humidity) without encapsulation, the D149-Cs_2_AgBiBr_6_@Ti_3_C_2_T_*x*_-based devices exhibited improved long-term stability with just 14% PCE loss. In Cs_2_AgBiBr_6_ devices, a photoactive dye called Zn-Chl was used as an HTL in addition to changing the ETL. The Zn-Chl-sensitized solar cell exhibited a PCE of 2.79% and *J*_SC_ of 3.83 mA cm^−2^, which is 22–27% greater than the devices using traditional hole transport materials (HTMs), such as spiro-OmeTAD, P3HT, and PTAA.^195^ Although this technique produced reasonably high *J*_SC_ and PCEs, the dyes rather than the Cs_2_AgBiBr_6_ absorber are responsible for the improvement.

An effective method to increase the inherent absorption capabilities of Cs_2_AgBiBr_6_ is element doping or alloying. A series of Cs_2_AgSb_*x*_Bi_(1−*x*)_Br_6_ (*x* = 0, 0.25, 0.50, and 0.75) thin films with progressively smaller bandgaps was produced by substituting SB^3+^ for Bi^3+^ in the structure. According to a study,^196^ the solar cell made utilizing a Cs_2_AgSb_0.25_Bi_0.75_Br_6_ thin film exhibited a clear enhancement in PCE compared to the solar cell used as a reference with Cs_2_AgBiBr_6_. However, following SB^3+^ alloying, the *J*_SC_ was reduced rather than increased, as anticipated, which is presumably because of the existence of big pinholes. An additional unique Cs_2_AgSbBr_6_ HDP could be created by completely substituting SB^3+^ for Bi^3+^. The Cs_2_AgSbBr_6_-based solar cells only produced a very low PCE of 0.01% with *V*_OC_ = 0.35 V, *J*_SC_ = 0.08 mA cm^−2^, and FF = 35.9% (ref. 197) due to the presence of impurity phases, as stated previously. Recently, the bandgap of the Cs_2_AgBiBr_6_ film was considerably reduced from 2.18 to 1.64 eV using a new hydrogen atom interstitial doping. With record PCE values of 5.64% and 6.37% for forward and backward scans, respectively, the *J*_SC_ of the solar cell impressively increased drastically from 1.03 to 11.4 mA cm^−2^.^198^ Upon treatment under nitrogen at 20 °C with light illumination, and 85 °C without or with light illumination for 1440 h, the devices retained almost 95%, 91%, and 84% of their original PCE, respectively. This indicates that the hydrogenated Cs_2_AgBiBr_6_ solar cells displayed good stability. Recently, the mixed-valence HDP MA_2_AuBr_6_ was employed as an absorber instead of Cs_2_AgBiBr_6_, although the device exhibited a very low PCE of 0.007%.^199^

**Fig. 15:**
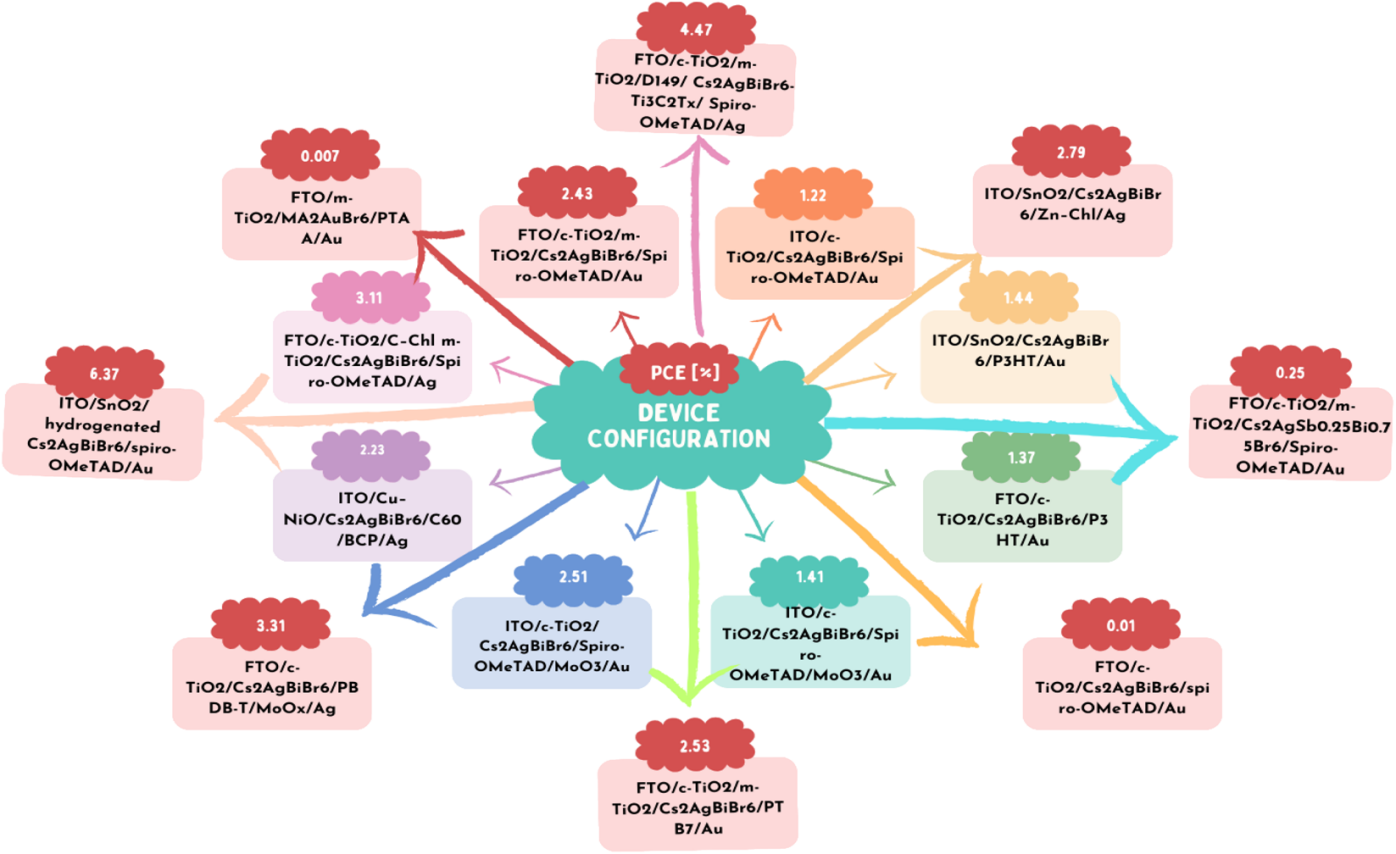
Device architecture and photovoltaic parameters of HDP-based solar cells.

In summary, lead-free halide double perovskite solar cells have emerged as a promising alternative to traditional Pb-based perovskites (Fig. 15), offering enhanced stability and reduced toxicity. The versatility in the choice of the B-site cations in HDPs opens up a plethora of possibilities for fine-tuning their properties, leading to a rich landscape of potential combinations and substitutional chemistry. The pioneering work on Cs_2_AgBiBr_6_ and subsequent innovations in deposition techniques, dye-sensitization, and elemental doping have paved the way for significant advancements in device efficiency and stability. However, although the achievements to date are commendable, as summarized in Table 7, the journey of HDPs is still in its initial stages. Thus, the exploration of new combinations, improved fabrication techniques, and deeper understanding of the underlying mechanisms will be crucial in realizing the full potential of HDPs in the field of photovoltaics. As research continues to evolve, HDPs stand poised to redefine the future of sustainable and efficient solar energy solutions.

**Table 7:** Recent development in halide double perovskite solar cells (HDPs)

Device configuration	Fabrication method	Significance/improvements	References
FTO/c-TiO_2_/m-TiO_2_/Cs_2_AgBiBr_6_/spiro-OMeTAD/Au	One-step spin coating	First double perovskite solar cell devices	200
ITO/c-TiO_2_/Cs_2_AgBiBr_6_/spiro-OMeTAD/Au	One-step spin coating	Highly uniform and high-quality thin film	201
ITO/SnO_2_/Cs_2_AgBiBr_6_/P3HT/Au	One-step spin coating and low-pressure-assisted method	Explored mechanisms affecting PV performance	202
FTO/c-TiO_2_/Cs_2_AgBiBr_6_/P3HT/Au	Sequential vapor deposition	Produced stoichiometric Cs_2_AgBiBr_6_ double perovskite thin films	190
ITO/c-TiO_2_/Cs_2_AgBiBr_6_/spiro-OMeTAD/MoO_3_/Au	Sequential vapor deposition	Produced planar Cs_2_AgBiBr_6_ double perovskite thin films	191
ITO/c-TiO_2_/Cs_2_AgBiBr_6_/spiro-OMeTAD/MoO_3_/Au	One-step spin coating	Micro-sized grains and high crystallinity	191
ITO/Cu-NiO/Cs_2_AgBiBr_6_/C_60_/BCP/Ag	One-step spin coating with antisolvent	Ultrasmooth morphology, micro-sized grains, high crystallinity	192
FTO/c-TiO_2_/C-Chl m-TiO_2_/Cs_2_AgBiBr_6_/spiro-OMeTAD/Ag	One-step spin coating	Smooth morphology and high crystallinity	193
FTO/c-TiO_2_/m-TiO_2_/D149/Cs_2_AgBiBr_6_-Ti_3_C_2_T_*x*_/spiro-OMeTAD/Ag	One-step spin coating	Increased sunlight absorption between 500 and 650 nm	194
ITO/SnO_2_/Cs_2_AgBiBr_6_/Zn-Chl/Ag	One-step spin coating	Zn-Chl-sensitized solar cell	195
FTO/c-TiO_2_/m-TiO_2_/Cs_2_AgSb_0.25_Bi_0.75_Br_6_/spiro-OMeTAD/Au	Dip coating	Substituting SB^3+^ for Bi^3+^ in the structure	196
FTO/c-TiO_2_/Cs_2_AgBiBr_6_/spiro-OMeTAD/Au	One-step spin coating	Presence of large defects	197
FTO/c-TiO_2_/m-TiO_2_/Cs_2_AgBiBr_6_/PTB7/Au	One-step spin coating	Presence of impurity phases	203
FTO/c-TiO_2_/Cs_2_AgBiBr_6_/PBDB-T/MoO_*x*_/Ag	One-step spin coating with antisolvent		204
ITO/SnO_2_/hydrogenated Cs_2_AgBiBr_6_/spiro-OMeTAD/Au	One-step spin coating with antisolvent	Reduced bandgap from 2.18 to 1.64 eV	205
FTO/m-TiO_2_/MA_2_AuBr_6_/PTAA/Au	Reactive polyiodide melt	Used as an absorber in place of Cs_2_AgBiBr_6_	199

## Tandem solar cells

Photoactive layers having a low band gap cannot achieve large voltages compared to large band-gap materials, which can achieve high voltages. However, despite the high voltages achieved, the short circuit current of these materials is limited by their low energy photons, which leads to constraints in single junction solar cells (SJSCs). Accordingly, tandem solar cells can be used to overcome these constraints. This arrangement integrates two or more single-junction solar cells for better use of short-wavelength photons from the visible spectrum. When photons with a small wavelength are absorbed by the top cell (perovskite 1), which is made of a large bandgap semiconductor material, a photocurrent is produced. Wider-wavelength photons pass through the top cell and into the perovskite cell bottom cell, where they are effectively absorbed. In essence, the bottom cell may use photons that are sent through the top cell, reducing the heat loss in PV devices even further. By properly combining the separate cells in series and parallel, the produced photo-voltage and photo-current can be increased. Utilizing tandem PSCs, record efficiencies of 29% have been achieved. A record PCE of 29.52% was reported by Oxford PV for perovskite silicon tandem solar cells, outperforming silicon solar cells by a wide margin.^206^ The monolithic perovskite–silicon tandem solar cell, which was designed with sinusoidal nanotextures, is very important for the control of light in PV devices.^207^

**Fig. 16:**
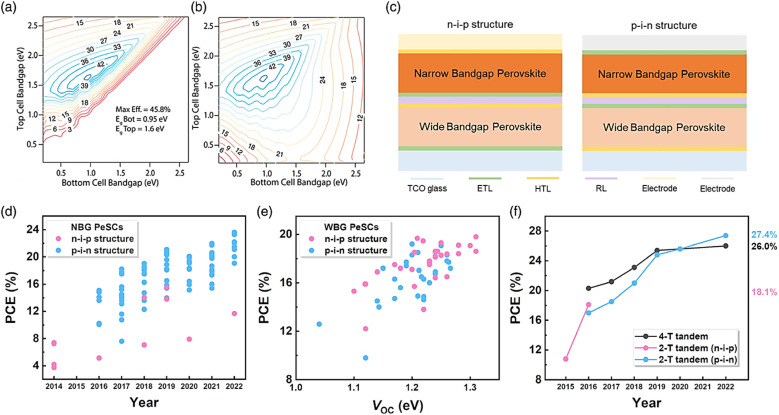
(a) Maximum efficiency limits for a 2-T tandem solar cell and (b) panel. (c) Configuration of a 2-T PTSC. (d) Efficiency of narrow bandgap (NBG) Sn–Pb PSCs with a bandgap range of 1.17–1.3 eV. (e) Efficiency and *V*_OC_ graph of large bandgap (WBG) Pb-based PSCs with a bandgap range of 1.7–1.8 eV. (f) PCE for 2-T and 4-T PTSCs over time. (a and b) Ref. 212.

The benefit of perovskite bandgap tuning is that it provides opportunities for use in tandem devices. The efficiency of two-bandgap tandem solar cells soared to a staggering 45%, surpassing their single-bandgap counterparts with a mere 33%. The theoretical bandgap diagram for tandem devices is shown in Fig. 16a.^208,209^ The combination of a 0.95 eV bottom cell and a 1.6 eV top cell yielded an incredible power conversion efficiency (PCE) of 45.8%, assuming the absolute absorption of sunlight. This can be achieved by changing the thickness of the light-absorbing layer. A regulated quantity of photons may be matched to the current of each device by adjusting the width of the broad bandgap front cell. As shown in Fig. 16b, the use of a large range of bandgap values was possible by current matching and combining two cells with bandgap values of 0.7–1.3 eV and 1.4–1.8 eV may provide a power conversion efficiency of 42%. The bandgap of all the Sn–Pb PSCs and Pb PSCs-based perovskite tandem solar cells (PTSCs) is around 1.2 and 1.8 eV, respectively.

The two-terminal (2-T) PTSC structure is schematically shown in Fig. 16c. 4-T PTSCs are mechanically arranged in series with 2-T PTSCs, which are merged monolithically by a recombination layer. For a series connection, the bottom and top cells should have identical polarity, which is described as either planer (n–i–p) or inverted (p–i–n) liable on the polarity. Given that PTSCs with p–i–n structures have received greater research attention, Sn–Pb PSCs with planer structures have a lower PCE than that with inverted structures (Fig. 16d). However, a larger power conversion efficiency and open circuit voltage in a planer structure are possible with Pb-based PSCs employing broad-bandgap cells (Fig. 16e). Researchers are examining Sn–Pb PSCs with a planar structure and Pb PSCs with an inverted structure, both of which have inferior performance to create a high-efficiency tandem device. The Grätzel group used chemical vapor deposition to manage the grain uniformity in the case of Sn–Pb PSCs with an n–i–p structure to generate big, uniform films for high efficiency, while the Hayase group added a passivation layer between the metal oxide and SnI_2_ to reduce the number of defects.^210,211^ In addition, p–i–n-structured PSCs have been investigated for use in tandem solar cells, and recently attained PCEs surpassing 26%.^212^

Although the efficiency of PTSCs is much lower than the theoretical value, they may still be used for a variety of applications, including water splitting.^213^ Water splitting is a procedure that uses incident photons on PTSCs to break down water into pure O_2_ and H_2_ and has lately gained greater attention as a subject in green hydrogen generation. Carbon-free methods such as thermoelectric, pyroelectric, triboelectric, and photoelectric power unlock the potential to produce hydrogen without pollution, paving the way for a sustainable future of limitless clean energy.^214^

The PEC system has a straightforward design and low device cost. However, it requires an external bias voltage and has a lower solar-to-hydrogen (STH) efficiency than PV-EC devices, making H_2_ production in PEC systems uneconomical.^215^ In contrast, a PV-EC system that separates the catalyst and battery may use two established technologies. Specifically, 1.23 V is the energy variance between former and latter to split water; however, a greater voltage is often needed to overcome the activation energy barrier. H_2_ is formed when a voltage higher than the activation energy is provided, and the quantity of hydrogen conversion is controlled by the current flow supplied by the PV component.

The Grätzel group demonstrated the first perovskite PV-EC. Two series-connected MAPbI_3_ solar cells were used to split water; however, because of the poor *J*_SC_, the STH efficiency was only 12.3%. They employed a silicon/perovskite tandem device rather than a series-connected device to boost the STH efficiency; consequently, the reduced *J*_SC_ loss permitted the STH efficiency of 18.7%.^216^ According to theoretical estimates, silicon/perovskite tandem cells for water splitting can attain a solar-to-hydrogen efficiency of 25% and a levelized hydrogen cost of less than 3 $ kg^−1^.^217^ With perovskite/silicon tandem solar cells, the STH efficiency has now reached 21.32%, causing us to assume that PTSCs have the potential to achieve STH efficiency levels higher than 20%. The best *V*_OC_ and *J*_SC_ of the obtained PTSCs are 2 V and 16 mA cm^−2^, respectively, when the performance of Sn–Pb PSCs increases.^218^ According to these *V*_OC_ and *J*_SC_ values, the STH efficiency of 19.68% can be attained, assuming that the voltage of the device is greater than the activation energy.^213^ It is anticipated that further study on Sn–Pb PSCs and PTSCs will lead to additional advancements in the device performance and STH efficiency.

**Fig. 17:**
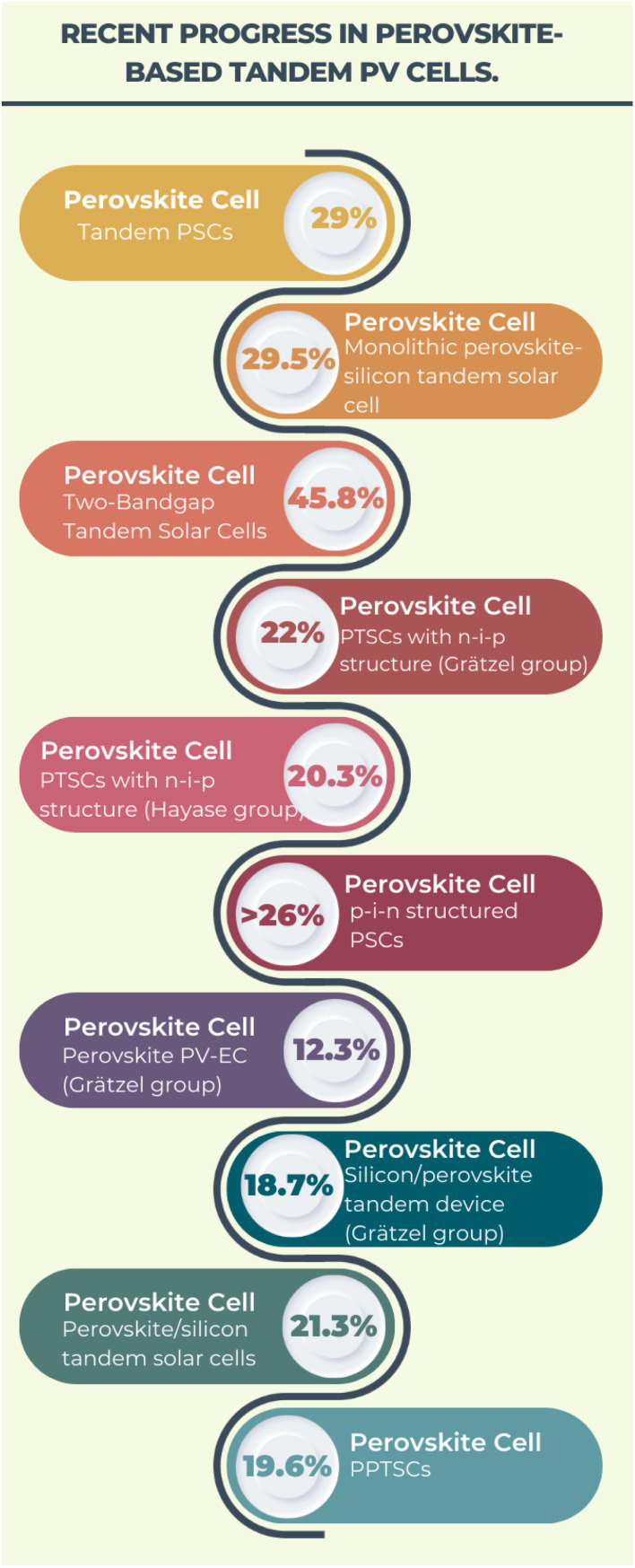
Recent progress in perovskite-based tandem PV cells.

In conclusion, tandem solar cells, particularly those based on perovskite technologies, represent a significant leap in the field of photovoltaics, as shown in Fig. 17. By harnessing the unique properties of perovskites and their ability for bandgap tuning, tandem configurations have successfully addressed the inherent limitations in single-junction solar cells. The advancements in PTSCs, not only in achieving record efficiencies but also in their potential applications such as water splitting, underscore their transformative potential in the renewable energy sector. The integration of perovskites with other materials, such as silicon, further amplifies the potential of these cells to achieve even higher efficiencies and broader applications. Table 8 shows a concise overview of the latest advancements in the performance of PTSCs. As research continues to push the boundaries of the possibilities with tandem configurations, it is evident that the future of solar energy is bright, with PTSCs poised to play a pivotal role in driving sustainable and efficient energy solutions for the world.

**Table 8:** A concise overview of the latest advancements in the performance of PTSCs

Structure	NBG *E*_g_ [eV]	NBG perovskite composition	WBG *E*_g_ [eV]	WBG perovskite composition	PCE [%]	Year	Ref.
4-T	1.24	FA_0.75_Cs_0.25_Sn_0.5_Pb_0.5_I_3_	1.8	FA_0.83_Cs_0.17_Pb(I_0.5_Br_0.5_)_3_	20.3	2016	219
	1.25	(FASnI_3_)_0.6_(MAPbI_3_)_0.4_	1.58	FA_0.3_MA_0.7_PbI_3_	21.2	2017	220
	1.25	(FASnI_3_)_0.6_(MAPbI_3_)_0.4_	1.75	FA_0.8_Cs_0.2_Pb(I_0.7_Br_0.3_)_3_	23.1	2018	221
	1.25	(FASnI_3_)_0.6_(MAPbI_3_)_0.4_	1.63	Cs_0.05_FA_0.8_MA_0.15_PbI_2.55_Br_0.45_	25.4	2019	222
	1.26	FA_0.7_MA_0.3_Pb_0.5_Sn_0.5_I_3_	1.59	FA_0.8_Cs_0.2_Pb(I_0.8_Br_0.2_)_3_	26.01	2022	223
2-T	1.55	MAPbBr_3_	2.3	MAPbI_3_	10.8	2015	224
(n–i–p)	1.55	FA_0.85_Cs_0.15_Pb(I_0.3_Br_0.7_)_3_	2	MAPbI_3_	18.1	2016	225
2-T	1.24	FA_0.75_Cs_0.25_Sn_0.5_Pb_0.5_I_3_	1.8	FA_0.83_Cs_0.17_Pb(I_0.5_Br_0.5_)_3_	17.0	2016	219
(p–i–n)	1.22	MAPb_0.5_Sn_0.5_I_3_	1.82	MA_0.9_Cs_0.1_Pb(I_0.6_Br_0.4_)_3_	18.4	2017	226
	1.25	(FASnI_3_)_0.6_(MAPbI_3_)_0.4_	1.75	FA_0.8_Cs_0.2_Pb(I_0.7_Br_0.3_)_3_	21.0	2018	227
	1.22	FA_0.7_MA_0.3_Pb_0.5_Sn_0.5_I_3_	1.77	Cs_0.2_FA_0.8_PbI_1.8_Br_1.2_	24.8	2019	228
	1.22	FA_0.7_MA_0.3_Pb_0.5_Sn_0.5_I_3_	1.77	Cs_0.2_FA_0.8_PbI_1.8_Br_1.2_	25.6	2020	229
	1.22	Cs_0.05_FA_0.7_MA_0.25_Pb_0.5_Sn_0.5_I_3_	1.79	Cs_0.2_FA_0.8_Pb(I_0.6_Br_0.4_)_3_	27.4	2022	230

## Challenges in perovskite solar cells

### Crystal structure

An ideal perovskite crystal structure typically exhibits a cubic configuration, composed of the ABX_3_ material combination, where ‘A’ represents a larger monovalent cation, ‘B’ indicates a smaller divalent cation, and ‘X’ represents a monovalent anion. The stability of a perovskite structure can be predicted using Goldschmidt's tolerance factor, ‘*t*’, which can be calculated as follows:
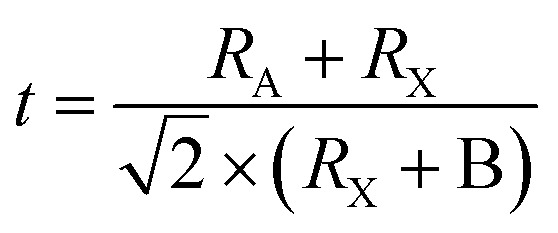
where *R*_A_, *R*_B_, and *R*_X_ are the ionic radii of the A, B, and X ions, respectively. The tolerance factor helps in assessing whether a particular combination of ions will form a stable perovskite structure. A stable perovskite crystal structure usually has a tolerance factor in the range of 0.7 to 1.0. Typically, a cubic structure is observed when the factor is in the range of 0.9 and 1. In this range, the ionic sizes are suitable for forming a cubic lattice, where the BX_6_ octahedra are undistorted, and the A-site cation fits snugly into the cuboctahedral void formed by the twelve X anions.^231,232^ Alternatively, tetragonal or hexagonal structures are formed when it exceeds 1. This usually indicates that the A-site cation is too large for the ideal cubic structure, leading to a distortion in the perovskite structure. Rhombohedral or orthorhombic structures are found in the range of 0.7 and 0.9. The A-site cation is relatively small, potentially leading to tilting of the BX_6_ octahedra. A tolerance factor below 0.7 usually indicates that the ionic radii are not conducive to forming a perovskite structure. The small size of the A-site cation compared to the B-site cation and X-site anion often prevents the formation of a stable perovskite lattice.

Accordingly, the tolerance factor of the cubic structure can be controlled, where altering the ionic radii of the A-site, B-site, or X-site ions by substituting them with different ions is a common method. For example, replacing a larger A-site ion with a smaller one (or *vice versa*) can lead to an increase or decrease in the tolerance factor. This substitution can be partial or complete, depending on the desired outcome.^233^

However, the perovskite crystals tend to have inherent defects, such as vacancies in lead (Pb) and iodine(i) ions, especially near the grain boundaries, which can compromise the stability and efficiency of PSCs. The presence of defects in perovskite crystals primarily affects their electronic properties by introducing trap states in their bandgap, which can capture charge carriers (electrons and holes), leading to non-radiative recombination. This process significantly reduces the efficiency of devices, given that it decreases the number of available charge carriers. Furthermore, defects can also aggravate ion migration, which not only contributes to hysteresis in the current–voltage characteristics of perovskite solar cells but also lead to long-term degradation of the material. This migration can alter the composition of the material over time, leading to phase instability and a decrease in device performance. Additionally, the presence of defects at the grain boundaries and interfaces can compromise the structural integrity of perovskite films, leading to mechanical weaknesses, which makes them more susceptible to environmental degradation factors such as moisture, oxygen, and heat. In solar cell applications, this degradation can manifest as a decline in power conversion efficiency and longevity of the device.^234^

To overcome the above-mentioned issues, doping with metal ions has been shown to be a successful approach.^235,236^ For instance, doping with monovalent metal cations such as lithium (Li^+^), copper (Cu^+^), and silver (Ag^+^) has been shown to decrease the density of trap states, enhance the crystallinity of the perovskite, and improve the quality of the film, leading to a better performance in PSCs.^237–239^ These improvements in the structural and electronic properties of the perovskite material directly contribute to the enhanced efficiency and stability of solar cells.

Additionally, doping with bivalent cations such as zinc (Zn^2+^), manganese (Mn^2+^), and cobalt (Co^2+^) has also been employed to increase the stability of PSCs. Trivalent metal cations such as indium (In^3+^), europium (Eu^3+^), and aluminum (Al^3+^) are frequently used to mitigate deep flaws, refine the film morphology, and boost both the efficiency and stability of PSCs.^233^ Notably, devices with Eu^3+^ doping have demonstrated remarkable endurance, maintaining 92% of their original PCE even after 1500 h of constant illumination. In a study at the University of Electronic Science and Technology in China, researchers have made substantial strides in enhancing the stability of perovskite solar cells (PSCs). By passivating grain boundary defects using a fluorinated oligomer derived from 4,4-bis(4-hydroxyphenyl) pentanoic acid (FO-19), they have significantly reduced the charge recombination, thus improving both the humidity and thermal stability of the cells. This approach led to an impressive PCE of 21.23% in MAPbI_3_-based PSCs.^234^ The coordination of the FO-19 carboxyl bond with Pb ions in the perovskite crystals effectively passivates defects, enhancing the overall stability of the perovskite film. This longevity is a significant step forward in the practical application of PSCs, making them more viable for long-term use in various settings.

### Stability

Perovskite solar cells (PSCs), while offering high power conversion efficiencies (PCE) and lower manufacturing costs compared to silicon solar cells, exhibit substantial stability issues, hindering their path to commercialization. Various degradation mechanisms, unique to each solar cell type, need to be addressed, particularly for PSCs. Factors such as moisture, oxygen, elevated temperatures, and UV illumination significantly affect their performance, necessitating specialized encapsulation strategies for protection.^232^

The core stability issues in PSCs originate from the inherent phase instability of the perovskite crystal structure and the design of the device itself. The key factors contributing to this instability include exposure to water (H_2_O), oxygen (O_2_), ultraviolet light, and heat.^240^ Methylammonium lead iodides, common components in PSCs, are particularly susceptible to hydrolysis when exposed to moisture. This process leads to material degradation, forming lead iodide (PbI_2_) and other by-products, which dramatically reduce the cell efficiency. Oxygen exposure can lead to oxidative degradation, especially under photo-induced conditions, forming reactive oxygen species that attack the perovskite structure. Similarly, UV light can degrade the organic components in the perovskite, creating defects and trap states, which diminish the cell efficiency. Additionally, the thermal instability due to high temperatures can cause phase segregation, introduce crystalline defects, and alter the material composition, further impacting the performance of the cell.

Encapsulation strategies play a crucial role in enhancing the lifespan of PSCs by acting as barriers against oxygen and moisture.^240^ Recent advancements include implementing glass-to-glass encapsulation, applying hydrophobic coatings, and replacing the reactive metal electrodes with more stable materials such as carbon and transparent conducting oxides. These methods effectively shield the perovskite from environmental stressors. For example, electron beam-deposited SiO_2_ layers in a glass cover have shown promising long-term stability. To further improve stability, researchers are exploring a variety of encapsulation materials, such as ethylene methyl acrylate, ethylene vinyl acetate, and polyisobutylene, with glass–polymer–glass structures proving particularly effective in preventing moisture ingress.

Addressing thermal instability is also crucial, given that the perovskite components can degrade at temperatures above 100 °C, forming more PbI_2_ and organic salts.^240^ Innovations such as replacing TiO_2_ with CdS as the electron transport layer and using carbon nanotubes wrapped with conductive polymers as the hole transport layer have shown potential in enhancing the thermal stability and improving the morphology of the device. However, even with these improvements, the maximum stable lifespan of PSCs under continuous light exposure typically reaches around 4000 h, with their efficiency declining as degradation progresses.

Photobleaching effects and the absence of encapsulation can cause further photo-instability, particularly in devices utilizing TiO_2_ layers, which are sensitive to UV light.^241^ In this case, implementing anti-UV coatings on the front glass and adding interlayers with high light transmission and electrical properties can significantly enhance the stability. These interlayers serve multiple functions, including suppressing charge recombination, modifying the perovskite surface, preventing moisture ingress, and blocking the diffusion of other materials.

Noteworthy research in this field includes the work by Henry J. Snaith's team,^242^ who investigated inverted perovskite solar cells using a copper phthalocyanine (CuPc) hole transport layer. This study, notable for its insights into long-term stability, showed that these solar cells demonstrated remarkable endurance. The cells maintained efficiency after more than 5000 h of storage and 3700 h at elevated temperatures of 85 °C in a nitrogen environment. The utilization of CuPc as a hole transport layer is particularly significant, highlighting the crucial role played by material selection in enhancing the performance and durability of perovskite solar cells. These findings not only underline the robustness of the devices but also their potential applicability in real-world settings, contributing valuable knowledge to the ongoing development of solar energy technologies. In another study, Zhou *et al.*^243^ explored the device configuration of ITO/HTL-Free/MAPbI_3_/C_60_/BCP/Ag. This perovskite solar cell demonstrated impressive stability, maintaining its performance after 1000 h of light soaking at the maximum power point (MPP) under continuous illumination, achieving the PCE of 16.9%. This configuration highlights the potential of HTL-free structures in achieving stable and efficient PSCs. Similarly, Xie *et al.*^244^ focused on FTO/NiMgLiOx/FAMAPbI_3_/PCBM/Ti(Nb)Ox/Ag. The cell showed a notable PCE of 20.6% and maintained its stability for 500 h under continuous light soaking at MPP. The use of NiMgLiOx as an interlayer in this structure suggests its effectiveness in enhancing both the efficiency and stability.

Arora *et al.*^245^ focused on a device with FTO/meso-TiO_2_/CsFAMAPbI_3_-xBrx/CuSCN-rGO/Au. The solar cell experienced only a 5% drop in PCE over more than 1000 h at MPPT, achieving an efficiency of 20.2%. The inclusion of reduced graphene oxide (rGO) and copper thiocyanate (CuSCN) indicated their roles in prolonging the operational life of the cell. In another study, Jung *et al.*^246^ studied FTO/c-TiO_2_/m-TiO_2_/(FAPbI_3_)_0.95_(MAPbBr_3_)_0.05_/WOx/spiro/Au. This cell demonstrated a 5% drop in PCE for over 1300 h at MPPT, with an ambient temperature of 25 °C and PCE of 22.7%. The use of a mixed halide perovskite layer and WOx as a buffer layer indicated their effective contribution to stability. Similarly, Akin *et al.*^247^ studied the device configuration of FTO/Ru-doped SnO_2_/perovskite/spiro-OMeTAD(Zn-TPP)/Au, which showed exceptional stability, with only a 3% decrease in efficiency over 2000 h, achieving a PCE of 21.8%. The use of Ru-doped SnO_2_ and Zn-TPP incorporated in spiro-OMeTAD as a modified hole transport layer underscores the potential of doping and molecular engineering in enhancing the stability and efficiency of PSCs.

Further advancements in perovskite solar cell technology are evident in the development of perovskite/silicon tandem solar cells. Using an evaporation–solution combination technique, the research team led by Li *et al.*^248^ successfully fabricated a p–i–n type perovskite layer atop a fully textured silicon cell, achieving a PCE of 27.48%. Remarkably, these tandem cells demonstrated stability in nitrogen for over 10 000 h, showcasing a viable solution to overcome the efficiency and stability limitations commonly associated with single-junction perovskite cells. Table 9 shows the impact of various PSC device configurations on the stability and PCE.

**Table 9:** Impact of various PSC device configuration on stability and PCE

S#	Device configuration	Stability	PCE (%)	References
1	ITO/HTL-free/MAPbI3/C60/BCP/Ag	After 1000 h, 93% PCE was retained	16.9	243
2	FTO/NiMgLiO_*x*_/FAMAPbI_3_/PCBM/Ti(Nb)Ox/Ag	After 500 h in ambient conditions, the encapsulated device efficiency dropped by 15%	20.6	244
		After 500 h of thermal stress at 85 °C in the dark, the PCE drop was less than 10%		
3	FTO/meso-TiO_2_/CsFAMAPbI_3−*x*_Brx/CuSCN-rGO/Au	After 1000 h at 60 °C PCE drop of 5%	20.2	245
4	FTO/c-TiO_2_/m-TiO_2_/(FAPbI_3_)_0.95_(MAPbBr_3_)_0.05_/WBH/P3HT/Au	After more than 1370 h at 25 °C, 5% PCE drop	22.7	246
5	FTO/Ru-doped SnO_2_/perovskite/spiro-OMeTAD(Zn-TFSI2)/Au	After more than 2000 h under ambient conditions, a drop of 3% PCE	21.8	247
6	FTO/TiO_2_(Na-TFSI)/(FAPbI_3_)_0.95_(MAPbBr_3_)_0.05_/spiro-OMeTAD(Na-TFSI)/Au	After 500 h at 45 °C, a drop of less than 20% in performance	22.4	249
7	ITO/NiO_*x*_/MAPbI_3_/ZnO/Al	After 60 days in air at room temperature, maintained 90% of its original efficiency	16.1	250
8	ITO/SnO_2_/MAPbI_3_/PTAA/Ag	Under continuous annealing at 85 °C in N_2_, maintained more than 85% efficiency	20.2	251
9	ITO/SnO_2_/FA_0.95_Cs_0.05_PbI_3_/spiro-OMeTAD/Au	After 2880 h in an ambient atmosphere, decrease of 8% in PCE	21.6	252
		After 120 h of irradiation at 100 mW cm^−2^, decrease of 14%		
10	FTO/compact-TiO_2_/CdS//MAPbI_3_/spiro-OMeTAD/Au	After 12 h of full sunlight illumination, retained 80% of its efficiency	9.9	253
11	ITO/P3CTN/(FAPbI_3_)_0.95_(MAPbBr_3_)_0.05_/TMTAIBL/PCBM/C60/TPBi/Cu	After 1000 h of continuous illumination at 60 °C, maintained 80% efficiency	19.2	254
12	FTO/mp-TiO_2_/CdS:Cd(SCN_2_H_4_)_2_Cl_2_/CH_3_NH_3_PbI_3_/spiro-OMeTAD/Au	After 240 h, retained 86.2% PCE	20.1	255
13	FA_0.83_Cs_0.17_PbI_3_/CuPc-HTL	After over 5000 h in storage and 3700 h under 85 °C in an N_2_ environment, retained more than 80%	13.9	242

However, despite these advancements, the overall lifetime of PSCs, which decreases significantly at a standard degradation rate of 25%, remains considerably shorter than that of crystalline silicon solar cells.^256^ Silicon solar cells have benefited from over five decades of research and development, leading to highly stable, efficient, and commercially viable solar technologies with lifespans nearing 30 years. Nevertheless, the rapid progress of perovskite solar cells within just a decade of research, achieving high PCEs, fuels optimism that ongoing research and technological advancements will eventually address the stability issues plaguing perovskite cells, paving the way for their wider adoption in sustainable energy applications.

### Perovskite fabrication

The fabrication of PSCs presents several challenges that are pivotal to their performance and commercial viability. One of the primary challenges is achieving uniform film formation. Perovskites are typically deposited as thin films, and inconsistencies during this process can lead to defects, such as pinholes and non-uniform crystal sizes, which significantly impact the efficiency and stability of the cells. Another hurdle is controlling the crystallization process. The crystallization rate and conditions determine the quality of the perovskite layer, with factors such as temperature, solvent choice, and deposition technique playing crucial roles. Too fast crystallization can lead to poor crystal formation, while too slow may result in large grains that affect the charge transport. Additionally, the sensitivity of perovskites to environmental factors such as moisture and oxygen during fabrication necessitates controlled atmospheric conditions, adding complexity and cost to the manufacturing process. This sensitivity also extends to the instability of perovskite materials under operational conditions, particularly for lead-based perovskites, which raises concerns about their long-term durability and environmental impact.^257^

The synthesis of hybrid organic–inorganic metal halide perovskite crystals, although based on stoichiometric reactions, involves a complex interplay among various process parameters that critically influence the quality of perovskite thin films. Research in this field has predominantly focused on refining these parameters such as the ratio of the precursors in solution, processing temperatures, and various fabrication techniques to optimize the formation of the perovskite layer. The quality of the perovskite film is paramount in determining the performance of PSCs, given that it directly impacts crucial factors such as the light absorption efficiency, charge recombination rates, and carrier diffusion lengths. Consequently, enhancing the quality of the perovskite film is a key strategy in improving the overall performance of PSCs.

In the development of high-quality perovskite films, several synthesis factors must be meticulously controlled. These include the temperature at which the process is conducted, the concentration of solutions, the choice of precursors and solvents, the use of surfactants, the ambient atmosphere during synthesis, the duration of the process, and the rates of flow and distribution of materials. Each of these factors can significantly affect the properties of the resultant perovskite films. Additionally, managing the growth of perovskites on various substrates is essential to produce films with desirable characteristics such as large grain size, high levels of crystallinity, and smooth surface morphology. In this context, the deposition method plays a crucial role, given that it directly influences the structural morphology of the perovskite layer. Techniques such as one-step deposition, two-step spin coating, two-source vapor deposition, sequential vapor deposition, and vapor-assisted solution deposition represent some of the diverse approaches utilized in the fabrication of perovskite thin films. Each of these deposition methods offers unique advantages and challenges, and the choice of method can be pivotal in achieving the desired film characteristics.

The majority of MAPbI_3_ films reported to date have been prepared using a two-step deposition technique, where the composition and concentration of the precursor solution are critical in the solution processing technique. The electronic structure of MAPbI_3_ exhibits a high degree of stoichiometric flexibility and surface defect tolerance during fabrication, making it relatively stable against a variety of compositional fluctuations. However, the optimal ratio of MAI to PbI_2_ in the precursor solution for enhancing the perovskite performance remains a subject of debate. Various mechanisms have been suggested to explain the notable improvements in perovskite materials. The precursor concentration plays a vital role in dictating the crystallinity, morphology, and colloidal nature of halide perovskites. Colloidal particles in the precursor act as nucleation points, influencing the quality of the resultant perovskite films. Researchers such as Hong, Xie, and Tian have experimented with MAPbI_3_ films under different conditions, adjusting the MAI and PbI_2_ ratios to either I-rich or Pb-rich environments. Their findings indicated that solvent engineering and stoichiometry modifications can significantly impact the efficiency, photo-stability, surface morphology, and coverage of MAPbI_3_ films.^258^

Additionally, introducing excess MAI in the precursor solution, particularly when combined with a Lewis acid-base adduct deposition technique, has been shown to effectively reduce the non-radiative recombination at the grain boundaries of the films.^259^ Chen *et al.*^260,261^ observed that the release of organic species during annealing enabled the presence of PbI_2_ phases at the perovskite grain boundaries, potentially enhancing the carrier behavior and stability. They also noted that DMF is an effective solvent for PbI_2_ and MAI, capable of controlling the crystallization speed and aiding the formation of compact perovskite films. Wieghold *et al.*^262^ highlighted that higher concentrations of precursors lead to the formation of larger, more oriented grains in MAPbI_3_ films. Additionally, the research by Park BW and others^263^ demonstrated the significance of excess lead iodide in the perovskite precursor solution for achieving over 20% power conversion efficiency, primarily by reducing the number of halide vacancies. These studies collectively contribute to a deeper understanding of the fabrication of perovskite solar cells, offering insights into how various factors influence the overall performance and efficiency of these promising photovoltaic materials.

Hasan *et al.* conducted a detailed investigation into the diffusion and crystallization of perovskite materials, particularly focusing on the interplay between PbI_2_ and MAI in the perovskite structure. Utilizing a synchrotron source-based XRD instrument, they examined the perovskite material at varying incidence angles and for different molar ratios of PbI_2_ to MAI. Their findings revealed that a 1 : 1 ratio of PbI_2_ to MAI facilitated the most effective interdiffusion of these components, leading to a fully converted perovskite material. This complete conversion was crucial for enhancing the chemical bonding and stability in the perovskite structure. Their XRD analysis demonstrated clear differences in the crystallization patterns of the perovskite films across various molar ratios and angles of incidence, offering valuable insights into the optimal conditions for achieving high-quality perovskite layers.^264–266^ In another significant study, Bahtiar *et al.*^267^ explored the fabrication of a perovskite solar cell (PSC) with the structure of FTO/PEDOT:PSS/CH_3_NH_3_PbI_3_ using a sequential deposition method. Their approach involved a meticulous two-step perovskite deposition process, which greatly influenced the performance and structural properties of the PSCs. Initially, a PbI_2_ precursor solution was prepared by dissolving 900 mg of PbI_2_ in 2 mL of DMF, followed by continuous stirring at 70 °C for 24 h. Subsequently, this solution was spin-coated over a PEDOT:PSS layer under varying conditions of rpm, annealing temperature, and time. Subsequently, an MAI precursor solution was prepared using 90 mg of MAI in 2 mL of IPA and spin-coated atop the PbI_2_ layer. This method, particularly the single-step spin coating, was found to enhance the surface morphology of PEDOT:PSS, reduce the number of pinholes, and consequently increase the power conversion efficiency. This study highlighted that specific conditions, such as a spin-coating rpm of 1000 for 20 s, annealing temperatures of 40 °C and 100 °C, and annealing times of 180 and 300 s, resulted in perovskite films with improved structural properties, including a pinhole-free surface and larger grain sizes exceeding 500 nm.

Minhuan Wang and colleagues conducted an insightful study comparing the one-step and two-step deposition methods for fabricating CH_3_NH_3_PbI_3_-based perovskite solar cells. Their research focused on analyzing how these methods influence the performance and structural integrity of the perovskite layer. In the one-step process, they prepared a perovskite precursor solution by combining PbI_2_ and MAI in a DMF + DMSO solution with a 1 : 4 volume ratio. Subsequently, this solution was directly spin-coated on a substrate at 3000 rpm for 50 s. In contrast, the two-step method involved first spin-coating PbI_2_ at 5000 rpm for 5 s, followed by applying the MAI solution at 500 rpm for 30 s, and then annealing the substrate at 150 °C for 20 min.^268,269^ Their X-ray diffraction (XRD) analysis revealed that the films fabricated using the two-step method exhibited distinct and pure perovskite peaks, indicating better crystallinity and phase purity compared to the one-step method, where the XRD peaks were less defined with evidence of degraded I_2_. Additionally, scanning electron microscopy (SEM) images corroborated that the two-step method resulted in superior structural properties in the perovskite layer. The two-step fabricated films showed no pinholes and dense coverage, which are crucial for minimizing the leakage current, and thus enhancing the power conversion efficiency (PCE) of the perovskite solar cells.

In the study by Liu *et al.* on perovskite solar cells with the device architecture of ITO/ZnO/CH_3_NH_3_PbI_3_/P_3_HT/Ag, they further delved on the impact of the perovskite layer thickness on PSC performance. They employed two-step spin coating and thermal deposition methods to fabricate the perovskite layer with a thicknesses in the range of 100 to 600 nm. Their findings indicated that the PCE increased with a layer thickness of up to 330 nm, beyond which an increase in thickness led to a decrease in efficiency. The optimal thickness of 330 nm yielded efficiencies of 1.3% for thermal deposition and 1.8% for sequential deposition.^263^ Notably, for thicker CH_3_NH_3_PbI_3_ films, the deposition of the P_3_HT polymer hole transport material faced challenges in adequately penetrating the perovskite layer, potentially leading to increased series resistance and decreased PCE.

These studies, together with numerous other reports on varying the precursor concentrations using different fabrication methods, highlight the critical importance of the deposition techniques and layer thickness in optimizing the performance of perovskite solar cells. The meticulous control of these factors is essential for enhancing the efficiency, stability, and overall viability of perovskite-based photovoltaic technologies. Table 10 presents the most common perovskite film fabrication techniques.

**Table 10:** Perovskite film fabrication techniques

S#	Methods	Description	Advantages	Disadvantages	References
1	One-step deposition	The creation of perovskite involves applying a mixture of organic and inorganic elements to a base surface leading to the concurrent crystallization of perovskite	Economical and straightforward to execute, with rapid processing times. Ideal for mass production	The efficiency is hampered by substandard film quality, and the requirement for a controlled…	270
2	Two-step spin coating deposition	A two-phase procedure is employed, where initially a solution with inorganic components is spin-coated, followed by the addition of an organic component	Offers enhanced control over the crystal formation process, leading to improved material quality	The process is time-consuming and sometimes results in residual PbI_2_	271
3	Sequential vapour deposition	This method involves first forming a bi-layer film of inorganic and organic components, which then transforms into perovskite	Addresses the limitations of one-step deposition by offering a more uniform film formation	Energy-intensive due to the vacuum process, and its scalability is limited	272
4	Two-source vapour deposition	In this technique, organic and inorganic materials are simultaneously evaporated to form perovskite	Leads to higher efficiency due to improved film uniformity	Energy consumption is high, and the process requires precise control	273
5	Vapour-assisted solution deposition	This process combines spin-coating of inorganic components with subsequent vapor treatment to form perovskite	Utilizes the benefits of both vapor and solution-based methods for better film quality	The requirement for a vacuum process makes it energy-intensive	274

### Band gap alignment & band offsets

Developing efficient electron and hole transport layers (ETLs and HTLs) with compatible energy levels is essential for creating high-efficiency and stable PSCs. The electrical and optical properties of perovskites are significantly influenced by their band structure, which is determined by the quantum mechanical wave functions permissible in the crystal. The bandgap of the materials used in PSCs is particularly crucial for effective visible light absorption, reducing the capacitive effects at the interfaces and maintaining the device stability. A notable aspect is the impact of the large bandgap of the ETL, which can restrict light absorption. Similarly, the matching of band structures in PSCs is a key factor in the rapid separation of electrons and holes, which aids in quickly dissipating capacitive charges and minimizing the hysteresis effect. The degree of conduction band alignment between the perovskite material and the ETL dictates the flow of electrons. Ideal alignment ensures smooth flow of charge carriers, while any offset can create barriers, hindering this flow. Similarly, the alignment of the valence bands between the perovskite and the HTL is crucial for efficient hole transport. Achieving high operational efficiency in PSCs requires the minimum conduction band offset (CBO) and maximum valence band offset (VBO) at the perovskite–ETL interface. This alignment allows for the efficient transfer of electrons from the perovskite to the ETL, while blocking holes. Conversely, the interface between the perovskite and HTL should aim for minimal VBO and maximal CBO, facilitating the movement of holes to the HTL and blocking electrons.^275^

When the conduction band (CB) of the perovskite layer is higher than the CB of the ETL (creating a cliff, or negative CBO), it can adversely affect the performance of the PSC by reducing the activation energy against recombination at the heterojunction and lowering the built-in potential (Vbi), which results in a decreased open-circuit voltage (*V*_oc_). In contrast, if the CB of the perovskite is lower than the CB of the ETL (forming a spike, or positive CBO), it leads to an increased Vbi, enhancing the *V*_oc_. However, excessively large spikes can impede the electron transport, increasing the charge recombination due to the reduced activation energy. Similarly, the alignment of valence bands affects the hole transport. If the valence band (VB) of the perovskite layer is below the VB of the HTL, it creates a cliff-like discontinuity (negative VBO), reducing the Vbi and impacting efficiency. If the VB of the perovskite is above the VB of the HTL, a spike (positive VBO) occurs, increasing the Vbi. Similar to the CBO, overly large spikes in the VBO can create barriers to hole transport.^276^

Band gap engineering is a crucial aspect in the advancement of highly efficient perovskite solar cells (PSCs). For instance, standard anatase TiO_2_, with a band gap of 3.2 eV, can only absorb about 5% of solar energy, which limits its effectiveness in solar cell applications. Thus, to enhance UV-visible light photocatalysis and broaden the absorption range, researchers have investigated doping TiO_2_ with various metals (such as V, Fe, Cr, and Ni) and non-metals (such as S, F, C, N, and B). This doping not only improves the quality of the semiconductor material by expanding its absorption range but also increases the mobility of charge carriers.^277^ The charge separation and transportation in PSCs are significantly influenced by the energy band alignment and the built-in internal electrical field. Ming Wang *et al.*^278^ developed a perovskite solar cell with the configuration of ITO/PEDOT:PSS/MAPbI_3_-XClX/PCBM/Rhodamine/LiF/Ag to explore how band gap tuning in the perovskite layer leads to rapid hole extraction. They found that as the concentration of MAI increases, reducing the band gap of the material, the charge transportation is enhanced, thereby increasing the current density. With an MAI concentration of 4 mg mL^−1^, the *J*_sc_ increased to 23.52 mA cm^−2^, resulting in a high PCE of 16.67% in MAPbI_3_-xClx-based PSCs.

**Fig. 18:**
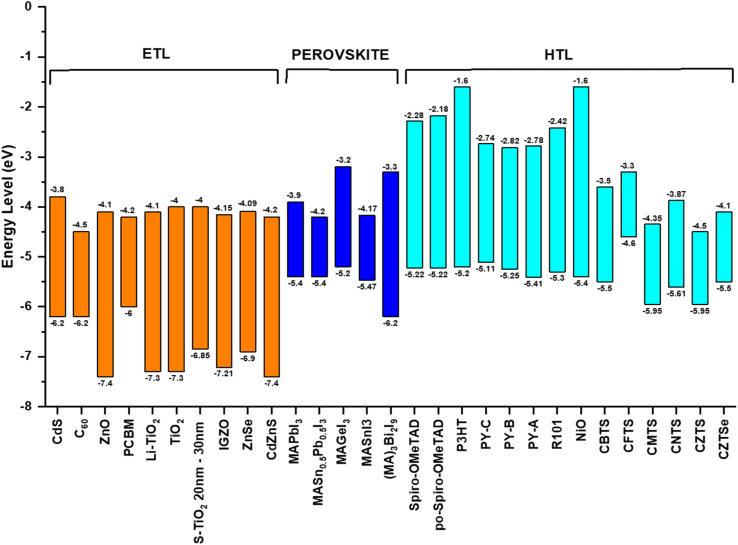
Energy levels of different materials used in PSCs.

Zhang *et al.*^279^ studied the effect of band gap tuning on the performance of perovskite solar cells by incorporating Sb in the CH_3_NH_3_PbI_3_ material. This adjustment regulated the band gap from 1.55 to 2.06 eV. A larger band gap was observed due to the reduced Pb bonding caused by the stronger interaction of Sb with the CH_3_NH_3_PbI_3_ material. The optimal Sb doping resulted in increased electron density in the conduction band and raised the quasi-Fermi energy level. Consequently, the built-in potential in the Sb-1%-doped cells increased, leading to a significant enhancement in *V*_oc_ and improved electron transport. The performance of the Sb-1%-doped solar cell outperformed the Sb-100%-doped cell, which is primarily because the *J*_sc_ in Sb-1% increased due to the longer charge diffusion length, ensuring efficient charge transport and collection. However, an increase in trap states in the Sb-100%-doped devices led to a degradation in *J*_sc_. Prasanna *et al.*^280,281^ delved into the impact of the band gap tuning of perovskite materials for solar photovoltaic applications. They highlighted that tin and lead iodide perovskite semiconductors are prominent candidates in PSCs partly due to their adjustable band gaps through compositional modification. Lead iodide-based perovskites exhibit an increase in band gap with the partial replacement of formamidinium and cesium due to octahedral tilting. Conversely, tin-based perovskites show a reduction in band gap without octahedral tilting. The band gaps achieved through this compositional tuning are ideal for tandem-based perovskite solar cells, capable of harvesting light up to approximately 1040 nm in the solar spectrum. This study underscores that ideal perovskite solar cells require specific material properties, such as a direct and suitable band gap, a sharp band edge, a long charge carrier lifespan, a long diffusion length, and a low exciton binding energy. Thus, band gap engineering strategies are vital for optimizing the energy band structures, significantly impacting the light harvesting and PCE. Fig. 18 show the energy levels of the different materials used in PSCs.

## Future opportunities

A promising avenue is the development of flexible and lightweight PSCs. The inherent properties of perovskite materials make them suitable for incorporation in flexible substrates, paving the way for a new generation of lightweight, portable, and even wearable solar-powered devices. This flexibility can revolutionize the integration of solar cells into everyday objects and structures, ranging from clothing and portable chargers to building facades and vehicles.

Building-integrated photovoltaics (BIPV) represents a growing sector, where perovskite solar cells (PSCs) can offer substantial advancements. The integration of PSCs in building materials, such as windows, facades, and roofing, aligns with the increasing demand for sustainable and energy-efficient building designs.^282–287^ The unique advantage of PSCs in BIPV applications originates from their high power conversion efficiency, lightweight nature, and potential for aesthetic integration. Unlike traditional photovoltaic systems, PSCs can be fabricated with varying colors and transparency levels, making them more architecturally versatile for integration into building surfaces without compromising the design aesthetics.

Furthermore, the potential of semi-transparent PSCs allows for their use in windows and glass facades, where they can generate electricity, while allowing some natural light to pass through. This dual functionality is particularly valuable in urban settings, where space constraints limit the installation of conventional solar panels. The use of PSCs in BIPV also contributes to reducing the heat gain inside buildings, potentially lowering cooling costs and enhancing the overall energy efficiency. In addition, the ease of manufacturing and the possibility of creating flexible perovskite modules extend the range of architectural applications. They can be integrated in curved surfaces and unconventional building shapes, opening new avenues for innovative and sustainable architectural designs. The incorporation of PSCs in BIPV systems can significantly contribute to the generation of renewable energy at the point of use, reducing the reliance on grid-supplied power and carbon footprint of buildings.

However, challenges such as ensuring long-term stability, weather resistance, and scalability of PSCs are areas of ongoing research. Addressing these challenges is crucial for the successful implementation of PSCs in BIPV, which holds the promise of transforming buildings from passive structures into active energy producers, aligning with global goals for sustainable development and energy efficiency.

## Conclusion and perspective

Perovskite solar cells (PSCs) have emerged as a promising contender in the field of photovoltaic technology, demonstrating rapid advancements in efficiency and adaptability. This review discussed these materials in-depth, highlighting their unique attributes and the challenges they present. The stability and efficiency of PSCs have been enhanced by techniques such creating electron and hole transport layers, using interfacial layers, and considering tandem solar cells. In-depth research has been done on lead-based, carbon-based, and tin-based PSCs, each of which has benefits and disadvantages. The investigation of tin- and carbon-based replacements has resulted from efforts to minimize or remove the lead concentration in PSCs. Additionally promising are polymer-based PSCs and lead-free halide double perovskite solar cells (HDPs). To promote the commercialization and broad acceptance of PSCs in industrial applications, future research should concentrate on improving the stability, scalability, and cost-effectiveness and creating lead-free perovskite materials and innovative ETLs. In the next years, PSCs are expected to make further strides and breakthroughs. Although significant development has been achieved, there are still several issues that need to be resolved before these solar cell technologies can reach their full potential.

Enhancing the stability and long-term performance of PSCs should be the main topic of future study. To ensure the viability and longevity of PSCs, efforts must be made to avoid moisture intrusion, enhance interfacial engineering, and create stable materials that can tolerate environmental variables. Additionally, lead-free alternatives should be further investigated, with an emphasis on creating effective and durable perovskite materials based on tin and carbon. The large-scale commercialization of PSCs necessitates the optimization of scalability and cost-effectiveness. PSCs will need to be more affordable to be more widely available, which will require streamlining manufacturing processes, improving deposition methods, and creating cost-effective materials. Device engineering and interface improvement will continue to be crucial in addition to material developments. Also, to improve the overall device performance, research should concentrate on creating new electron and hole transport layers, investigating alternate interfacial layers, and increasing charge extraction. The commercialization and broad acceptance of PSCs will be facilitated by the coordinated efforts of researchers, industry players, and policymakers. Gaining the full potential of PSCs in offering clean and sustainable energy solutions will be accelerated by continued investment in research and development, information exchange, and cooperation.

In conclusion, PSCs have enormous potential to revolutionize the solar energy conversion industry. Perovskite materials have several outstanding characteristics, and current research and development efforts are opening the door to extremely effective, inexpensive, and environmentally friendly solar cells. PSCs have the potential to change the clean energy landscape and contribute to a sustainable and environmentally friendly future with continuing improvements in stability, scalability, cost-effectiveness, and lead-free substitutes.

## Conflicts of interest

The authors declare that they have no competing interest with any party.

## Supplementary Material
